# Fine mapping and identification of two *NtTOM2A* homeologs responsible for tobacco mosaic virus replication in tobacco (*Nicotiana tabacum* L.)

**DOI:** 10.1186/s12870-024-04744-y

**Published:** 2024-01-24

**Authors:** Xuebo Wang, Zhan Shen, Caiyue Li, Yalin Bai, Yangyang Li, Wenhui Zhang, Zunqiang Li, Caihong Jiang, Lirui Cheng, Aiguo Yang, Dan Liu

**Affiliations:** 1 Key Laboratory for Tobacco Gene Resources, Tobacco Research Institute, Chinese Academy of Agricultural Sciences (CAAS), Qingdao, 266101 China; 2 Tobacco Science Research Institute of Guangdong Province, Shaoguan, Guangdong 512029 China; 3 Hunan Tobacco Research Institute, Changsha, 410004 China; 4 Linyi University, Linyi, 276000 Shandong China; 5 Philippine Christian University Center for International Education, Manila, 1004 Philippines; 6 Tobacco Research Institute of Mudanjiang, Harbin, 150076 China

**Keywords:** Tobacco, Tobacco mosaic virus, Map-based cloning, *Tobamovirus multiplication protein 2A*, Virus replication, Tobacco germplasms, Breeding

## Abstract

**Background:**

Tobacco mosaic virus (TMV) is a widely distributed viral disease that threatens many vegetables and horticultural species. Using the resistance gene *N* which induces a hypersensitivity reaction, is a common strategy for controlling this disease in tobacco (*Nicotiana tabacum* L*.*). However, *N* gene-mediated resistance has its limitations, consequently, identifying resistance genes from resistant germplasms and developing resistant cultivars is an ideal strategy for controlling the damage caused by TMV.

**Results:**

Here, we identified highly TMV-resistant tobacco germplasm, JT88, with markedly reduced viral accumulation following TMV infection. We mapped and cloned two *tobamovirus multiplication protein 2A* (*TOM2A*) homeologs responsible for TMV replication using an F_2_ population derived from a cross between the TMV-susceptible cultivar K326 and the TMV-resistant cultivar JT88. Clustered regularly interspaced short palindromic repeats (CRISPR)/CRISPR-associated protein 9 (CRISPR/Cas9)-mediated loss-of-function mutations of two *NtTOM2A* homeologs almost completely suppressed TMV replication; however, the single gene mutants showed symptoms similar to those of the wild type. Moreover, *NtTOM2A* natural mutations were rarely detected in 577 tobacco germplasms, and CRISPR/Cas9-mediated variation of NtTOM2A led to shortened plant height, these results indicating that the natural variations in *NtTOM2A* were rarely applied in tobacco breeding and the NtTOM2A maybe has an impact on growth and development.

**Conclusions:**

The two *NtTOM2A* homeologs are functionally redundant and negatively regulate TMV resistance. These results deepen our understanding of the molecular mechanisms underlying TMV resistance in tobacco and provide important information for the potential application of *NtTOM2A* in TMV resistance breeding.

**Supplementary Information:**

The online version contains supplementary material available at 10.1186/s12870-024-04744-y.

## Background

Tobacco mosaic virus (TMV), a typical member of the genus *Tobamovirus*, causes a viral disease that poses a huge threat to hundreds of plant species worldwide, including *Arabidopsis thaliana*, tobacco, tomato, and pepper [1–5]. It can cause leaf chlorosis and mosaic symptoms, leading to severe disease and substantial agricultural losses [6]. Identifying resistance genes and developing resistant cultivars is an ideal strategy for controlling the damage caused by TMV in crops.

During the process of coevolution with pathogens, plants evolved a complex immune system [7, 8], which comprises pattern-triggered immunity (PTI) and effector-triggered immunity (ETI) [9–12]. The PTI system can prevent further invasion of plant pathogens, but in many cases, the pathogens are able to use effectors to escape PTI [13, 14]. The ETI system is induced by the direct or indirect recognition of *Avr* genes by R proteins, which can induce an *R* gene resistance response [15]. The* N* gene, cloned from the wild tobacco species *Nicotiana glutinosa*, was the first identified *R* gene in tobacco, which encodes a typical Toll-interleukin-1 receptor/nucleotide-binding site/leucine-rice-repeat (TIR-NBS-LRR) protein. It confers high resistance towards TMV by inducing a hypersensitive reaction (HR) at infection sites, which interferes with viral movement in plant tissues [16, 17]. However, *N* gene-mediated resistance is remarkably temperature-sensitive, and therefore, HR cannot occur when the temperature exceeds 28℃, which leads to the systematical spreading of the virus [18]. Besides, RNA silencing, phytohormone, and resistance conferred by mutations in susceptibility genes are also effective antiviral strategy in plants [19–21].

Successful viral infection and symptom development require plant host factors for viral genome replication as well as for cell-to-cell and long-distance movement through the plant [22–27]. Altering these host factors could provide a broad-spectrum and durable antiviral strategy [28, 29] and has occasionally been shown to cause extreme resistance characterized by a lack of symptoms as well as limited or no pathogen replication and spread [30, 31]. Several host genes responsible for TMV replication and movement have been identified in plants. Tobamovirus multiplication proteins are critical host factors for TMV replication [32–34]. In *A. thaliana*, *tobamovirus multiplication 1* (*TOM1*) and its homologous gene *tobamovirus multiplication 3* (*TOM3*) were shown to be responsible for the efficient replication of the crucifer-infecting tobacco mosaic virus (TMV-Cg) [35, 36]. Another *AtTOM1* homolog *THH1* was also shown to be involved in tobamovirus multiplication [37]. As a member of the replication complex, *AtTOM2A* encodes a transmembrane protein that interacts with *AtTOM1* and itself, playing a vital role in assisting TMV replication [38, 39]. In *Capsicum annuum*, the replication of TMV was suppressed by inhibiting the expression of *CaTOM1* and *CaTOM3* [5]. In tobacco (*N. tabacum* L*.*), the simultaneous silencing *NtTOM1* and *NtTOM3* effectively inhibited TMV replication [33]. Recessive mutations in *NtTOM2A* also significantly suppress TMV replication in tobacco [39]. Translation elongation factor 1A (eEF1A) and translation elongation factor 1B (eEF1B) are essential host factors for TMV infection, and eEF1B may be a component of the TMV replication complex that interacts with the MT domains of the TMV RdRp and eEF1A [40, 41]. Transcription factor *WRKY8* functions in TMV-Cg long-distance movement by mediating both abscisic acid and ethylene signaling in *Arabidopsis thaliana* [42]. The microtubule-associated plant factor *MPB2C* is required for the microtubular accumulation of the TMV movement protein (MP) in plants [23], and PAP85, a vicilin-like seed storage protein, was reported to be involved in TMV replication, as TMV accumulation was reduced in PAP85-knockdown protoplasts [43].

Tobacco (*Nicotiana tabacum* L*.*) is an allotetraploid plant species derived from primary hybridization between *Nicotiana sylvestris* (SS genome) and *Nicotiana tomentosiformis* (TT genome), followed by chromosome doubling. Thus, it has a relatively large (approximately 4.4 GB) and complex genome [44], which makes map-based cloning of tobacco genes difficult. In recent years, with the development of high-throughput sequencing, mapping of genes from tobacco germplasm resources has become feasible [39]. In the present study, we mapped two *NtTOM2A* homeologs from the tobacco germplasm JT88 using bulk segregant analysis (BSA) and map-based cloning. Double mutants that showed full loss-of-function in both homeologs showed high resistance to TMV. We also determined the distribution of the *NtTOM2A* allelic variations in 577 tobacco germplasm accessions. Our results demonstrate the significant role of *NtTOM2A* and its potential value in TMV resistance breeding.

## Results

### Phenotypic characterization under TMV-U1 infection

**Fig. 1:**
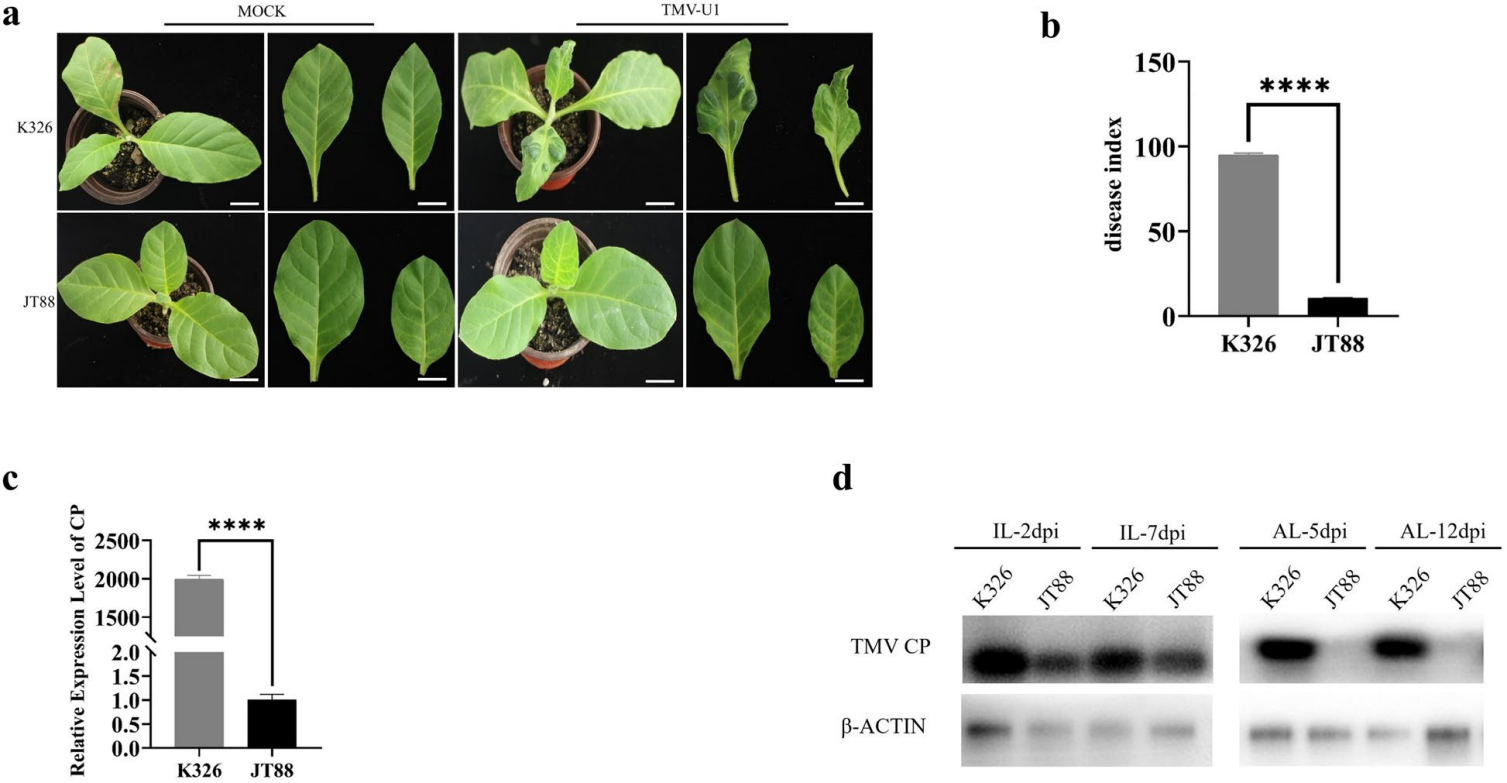
Phenotypic characterization of two different cultivars that responded to tobacco mosaic virus (TMV) infection. **a** Plant phenotype of the susceptible cultivar K326 and resistant cultivar JT88 after infection with TMV-U1 and phosphate buffer saline (MOCK), respectively. Scale bar = 2 cm. **b** Disease index (DI) of the susceptible cultivar K326 and resistant cultivar JT88 investigated at 14 dpi. **c** TMV CP relative expression levels in apical non-inoculated leaves (AL) of two different cultivars detected by qRT-PCR. The tobacco *actin* gene used as an internal reference gene. **d** TMV accumulation levels in the inoculated leaves (IL) and apical non-inoculated leaves (AL). Virus contents detected by Western blot with an anti-TMV coat protein antibody. The target blot was cropped from the full-length original blot. dpi: days post inoculation, MOCK: mock-inoculated with phosphate buffer saline, TMV CP: TMV coat protein, β-ACTIN: anti-plant actin mouse monoclonal antibody. The data (Fig. 1b and 1c) are presented as means ± SDs of three biological replicates. Student *t*-tests were used to determine the significance of differences between K326 and JT88 (****P < 0.0001)

The tobacco genotypes K326 and JT88 displayed marked differences in resistance to TMV. The genotype K326 developed a typical mosaic phenotype after inoculation with TMV-U1. In contrast, the JT88 genotype was highly resistant to TMV (Fig. 1a). The disease index (DI) of K326 was close to 100, whereas that of JT88 was approximately 10.8, indicating significantly differing resistance towards TMV-U1 (Fig. 1b). To confirm whether the TMV resistance of JT88 was related to the *N* gene, TMV resistance was determined using the *N*-marker [45]. ZY300, a tobacco cultivar harboring the *N* gene, exhibited HR after inoculation with TMV. However, no bands were amplified using the *N*-marker in JT88, and no HR was observed in the inoculated leaves of JT88 (Supplementary Material l Fig. S1a, b; Supplementary Material 2). These results indicated that the mechanism underlying the TMV resistance of JT88 was distinct from the resistance mediated by the *N* gene. Furthermore, the quantitative reverse transcription polymerase chain reaction (qRT-PCR) experiments indicated that compared to K326, JT88 had a lower TMV coat protein (CP) transcriptional level in the apical non-inoculated leaves (AL) (Fig. 1c). The western blot (WB) analysis showed that the CP protein content in the inoculated leaves (IL) of JT88 at 2- and 7days post inoculation (dpi) was lower than that of K326, and the CP protein content in the upper systemic leaves of JT88 at 5 and 12 dpi was almost undetectable (Fig. 1d; Supplementary Material 2). These results indicated that the replication of TMV in JT88 was suppressed.

### The candidate gene is located on chromosome Hic_asm_21

**Fig. 2:**
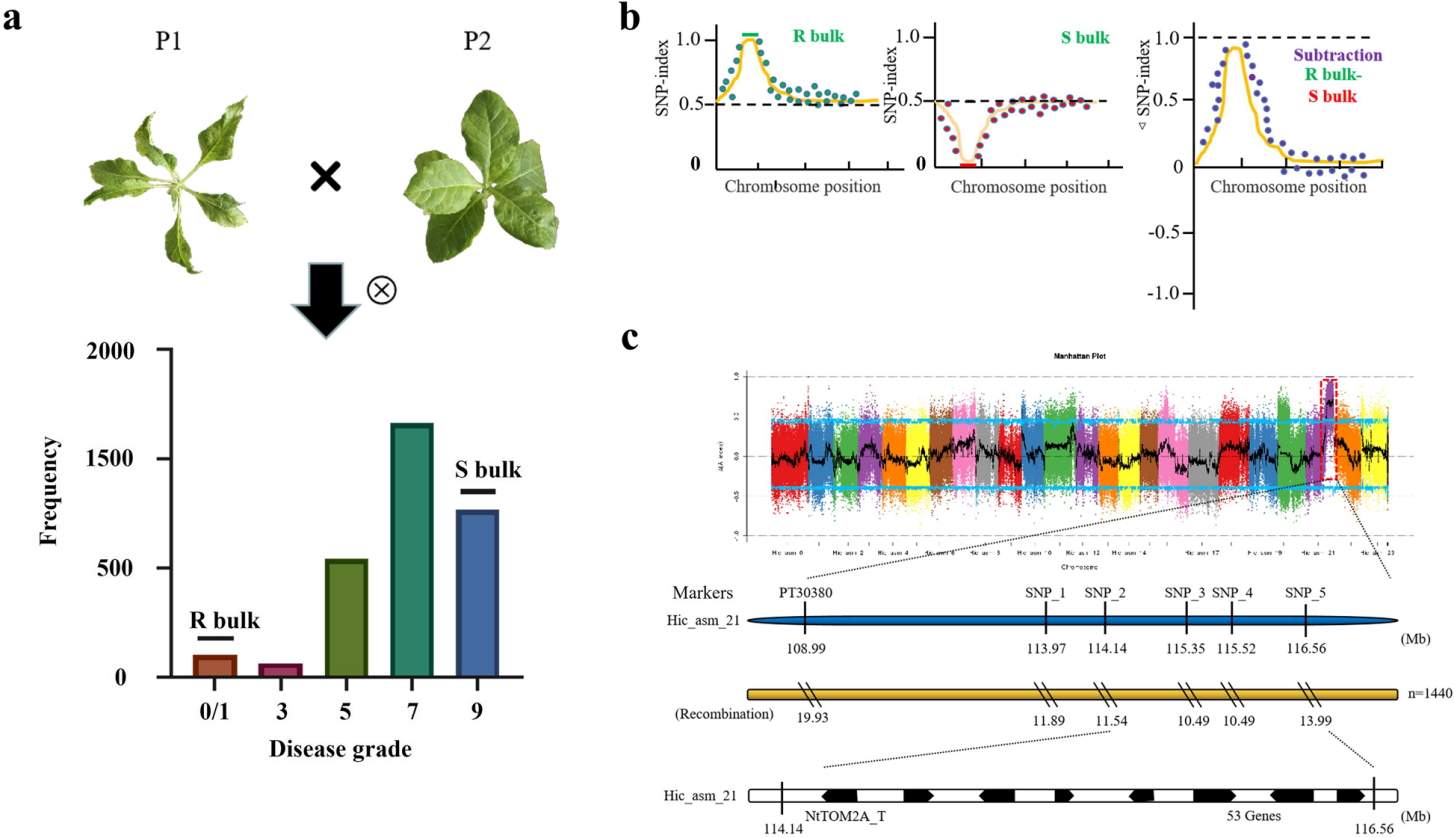
Genetic mapping of the *NtTOM2A_T* homeolog by bulked segregant analysis and map-based cloning. **a**,** b** Diagrammatic sketch of the bulked segregant analysis used in this research. P1: K326, P2: JT88. Numbers 0, 1, 3, 5, 7, and 9 represent the disease grade from the lowest to the highest. R bulk: resistant bulk, S bulk: susceptible bulk. Frequency: the corresponding amounts of different disease grade individual plants. **c** Genetic mapping of the *NtTOM2A_T* homeolog. Manhattan plot of the bulked segregant analysis was used for fine mapping the *NtTOM2A_T* homeolog, which was mapped to a 2.42 Mb region between SNP-2 and SNP-5 on chromosome Hic_asm_21 based on the crossover rate. This region contains 53 predicted genes

To identify the genomic regions associated with TMV resistance in JT88, an F_2_ population was constructed by crossing K326 and JT88, followed by selfing. After inoculation with TMV-U1, the mosaic symptoms of the F_2_ generations showed varying degrees of segregation (Supplementary Material l Fig. S2). Given the relatively large genome size and low polymorphism of *N. tabacum*, bulk segregant analysis (BSA) was used to obtain the approximate position of the resistance gene (Fig. 2a, b). A total of 627 M and 1,266 M reads were generated from the two parents and two bulks, respectively. The average sequencing depth of the parents was 9.5 × , while that of the bulks was 18.5 × (Supplementary Table S1, Supplementary Materia l Fig. S3a). About 93% and 98% of the whole genome in the bulks and parents, respectively, were covered by more than 4 × reads. In total, 705,477 single-nucleotide polymorphisms (SNPs) were used to calculate the SNP- index and ΔSNP-index (Supplementary Table S2). Based on the ΔSNP-index, a major region on the chromosome Hic_asm_21:105,906,001–145,206,000 was identified, in which the ΔSNP-index was close to 1.0 at a confidence level of 99% (Fig. 2c, Supplementary Material l Fig. S3b). The physical interval was approximately 39 Mb. Results of the BSA-Seq revealed a major candidate region for tobacco TMV resistance on chromosome Hic_asm_21.

### Fine mapping of the candidate gene

Among the simple sequence repeat (SSR) markers detected [46], PT30380 was found to be a co-dominant marker (Supplementary Material l Fig. S3c; Supplementary Material 2). Subsequently, 49 SNP markers in the 39 Mb candidate interval were developed based on the sequence differences between the two parents (Supplementary Table S3). Eventually, 28 SNPs were detected between the parents and their F_1_ generation within the interval. Five SNP markers and the SSR marker PT30380 were then selected to genotype 1440 F_2_ individuals. By calculating the genetic crossover rate of each molecular marker with the candidate gene, the crossover rates of PT30380, SNP-1, SNP-2, SNP-3, SNP-4, and SNP-5 with the candidate gene were determined to be 19.93, 11.89, 11.54, 10.49, 10.49, and 13.99, respectively. The resistance gene was located between SNP-2 and SNP-5, corresponding to the physical locations of 114.14 Mb and 116.56 Mb (physical distance 2.42 Mb) on the reference genome chromosome Hic_asm_21 (Fig. 2c).

### Identification of *NtTOM2A_T* and *NtTOM2A_S* homeologs

**Fig. 3:**
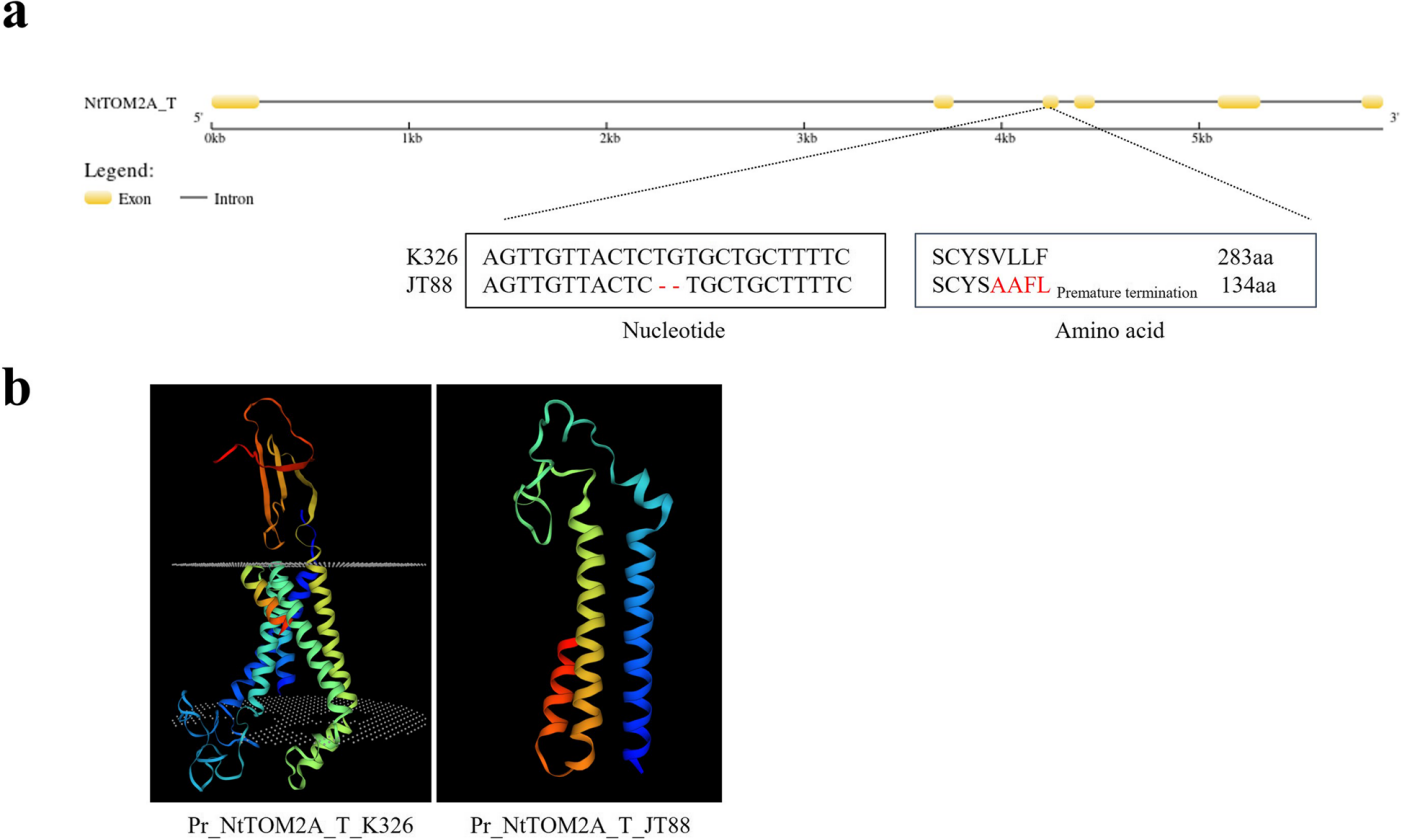
Identification of the *NtTOM2A_T* homeolog. **a** Gene structure of *NtTOM2A_T*, with the 2 bp deletion in JT88 marked by ‘-’, and the deduced amino acid sequence and final amino acid numbers marked by highlighting. **b** Predicted protein three-dimensional structure of NtTOM2A_T in K326 (left) and JT88 (right). bp: base pairs

A total of 53 predicted genes were identified in this physical interval (Supplementary Table S4). Among these genes, *evm.TU.Hic_asm_21.3347* was predicted to encode the *Tobamovirus multiplication protein 2A*, which is associated with TMV replication. The replication of TMV genomic RNA was found to be suppressed in the *tom2a* mutant, resulting in an asymptomatic phenotype upon TMV infection [37]. Sequence analysis showed that the *evm.TU.Hic_asm_21.3347* gene derived from *Nicotiana tomentosiformis* subgenome (Supplementary Material l Fig. S4a, b). Thus, we renamed the *evm.TU.Hic_asm_21.3347* gene as *NtTOM2A_T*. The *NtTOM2A_T* gene sequence analysis of K326 and JT88 showed that the *NtTOM2A_T* gene of JT88 had a 2 bp deletion in the third exon (Fig. 3a), which led to a frameshift mutation and resulted in premature termination of the amino acid sequence (Fig. 3b and Supplementary Material l Fig. S5a, b).

**Fig. 4:**
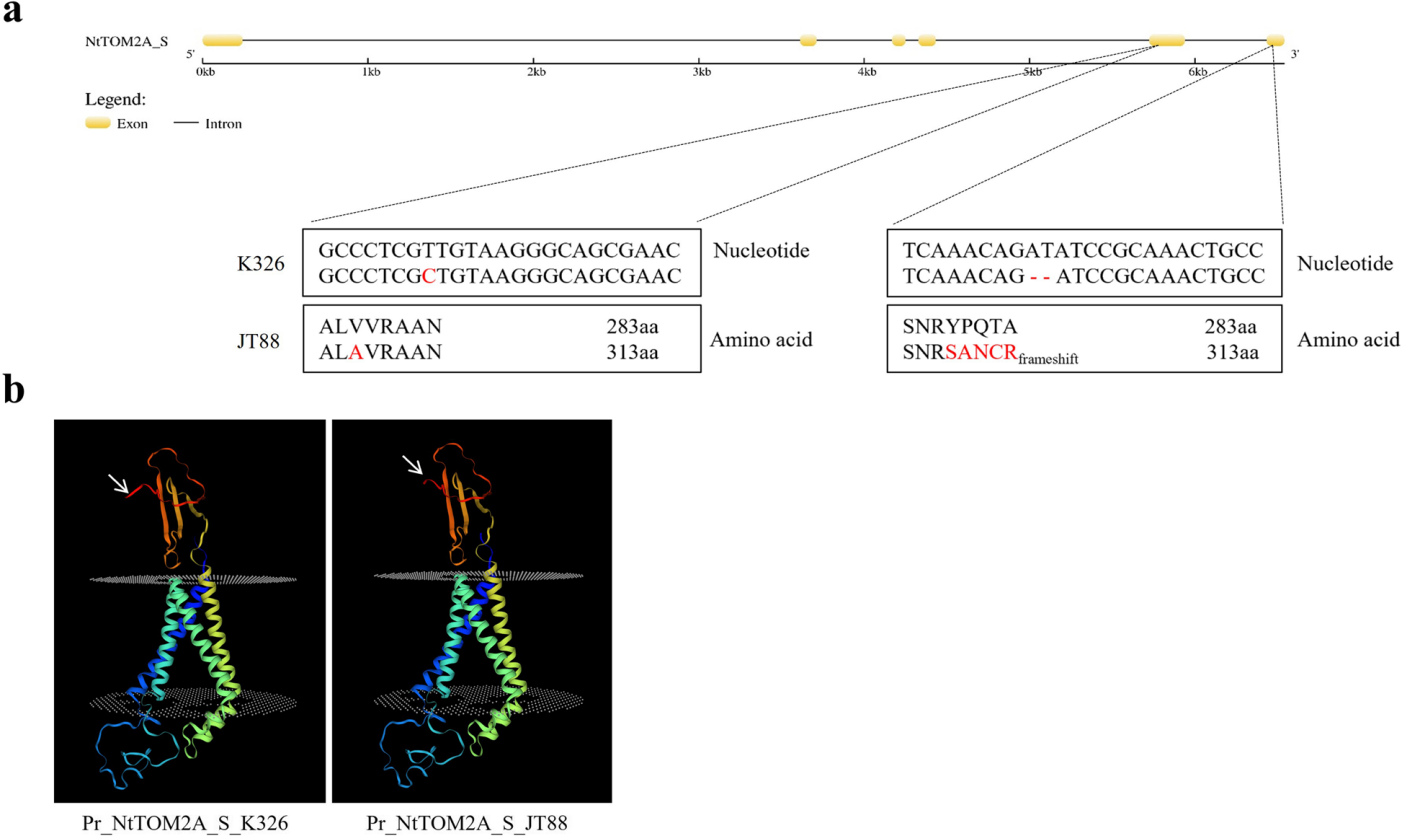
Identification of the *NtTOM2A_S* homeolog. **a** Gene structure of *NtTOM2A_S*, with the point mutation highlighted in red, the 2-bp deletion in JT88 marked by ‘-’, and the deduced amino acid sequence and final amino acid numbers marked by highlighting. **b** Predicted protein three-dimensional structure of NtTOM2A_S in K326 (left) and JT88 (right). bp: base pairs

As tobacco is allotetraploid, a previous study showed that the *TOM2A* homeolog derived from the *Nicotiana sylvestris* subgenome also contributes to its resistance to TMV [39]. In the present study, we designated this homologous gene as *NtTOM2A_S* based on its evolutionary relationship (Supplementary Material l Fig. S4a, b) and analyzed the *NtTOM2A_S* sequences in K326 and JT88, and the results showed that the *NtTOM2A_S* homeolog of JT88 had an SNP in the fifth exon and a 2 bp deletion in the sixth exon (Fig. 4a and Supplementary Material l Fig. S6a, b), which could change the protein’s three-dimensional structure (Fig. 4b). Based on these results, we identified *NtTOM2A_T* and *NtTOM2A_S* as the candidate genes. Sequence analysis showed that both homeologs contained four transmembrane regions (Supplementary Material l Fig. S4c).

### Loss-of-function of *NtTOM2A_T* and *NtTOM2A_S* confers TMV resistance in tobacco

**Fig. 5:**
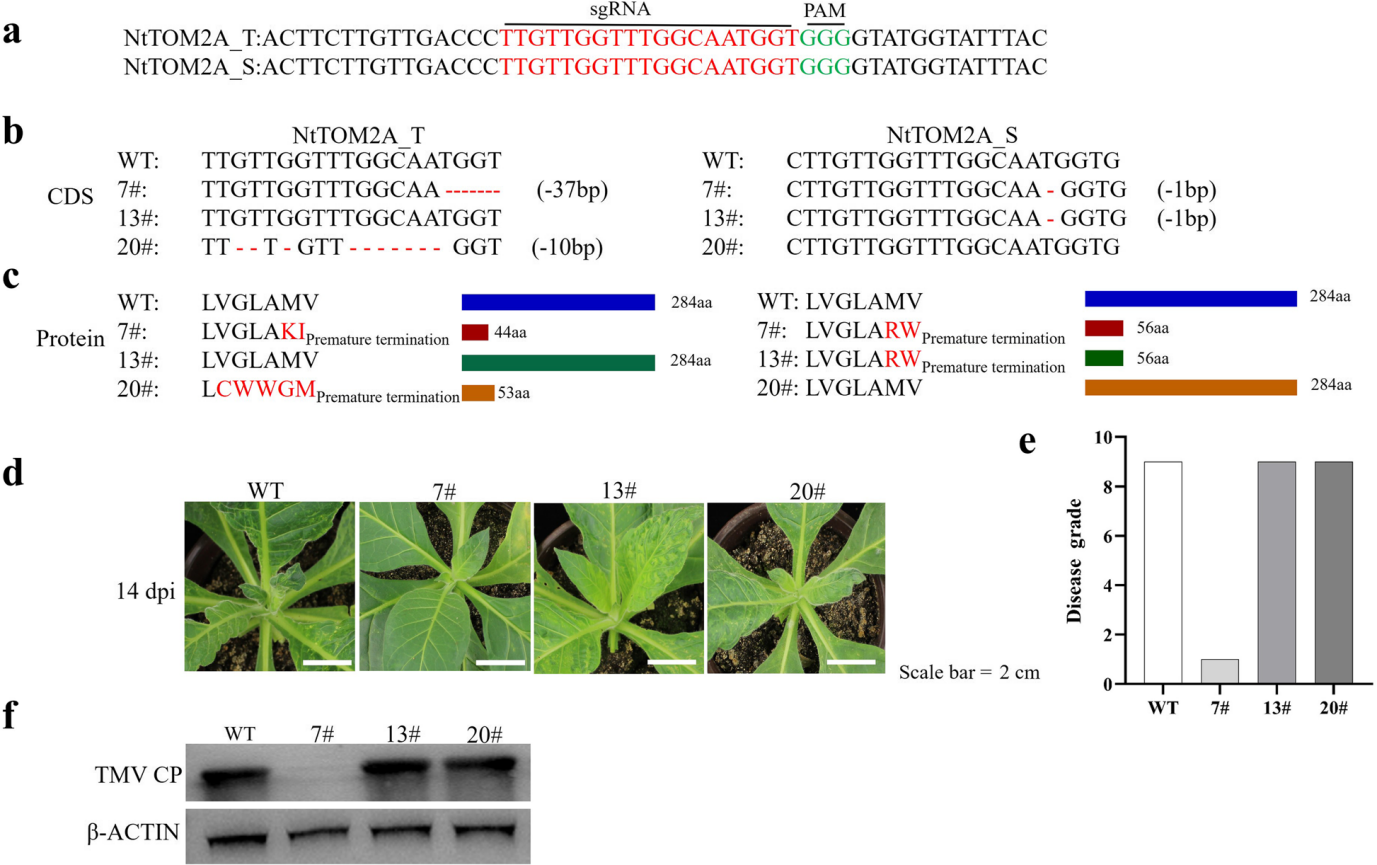
Functional identification of NtTOM2A under TMV infection. **a** The sgRNA sequence is highlighted in red and the protospacer-adjacent motif (PAM) is highlighted in green. **b**,** c** The NtTOM2A_T and NtTOM2A_S mutation sites of three different mutants and their corresponding amino acid sequences. **d** Phenotypes of three different mutants 14 days after infection with TMV. **e** The disease grade of wild type and three different mutants.** f** Virus accumulation in the apical non-inoculated leaves of the three mutants and wild type. Virus contents were detected by Western blot using an anti-TMV coat protein antibody. The target blot was cropped from the full-length original blot. β-ACTIN: anti-plant actin mouse monoclonal antibody

To verify whether these two homeologs are responsible for disease resistance, we employed the CRISPR/Cas9 system to identify their functions upon TMV infection. To mutate *NtTOM2A_T* and *NtTOM2A_S*, we designed one small guide RNA (sgRNA) targeting two homeologs in the K326 genotype using the CRISPR MultiTargeter (Fig. 5a) [47]. Finally, we obtained three types of homozygous mutants in the T_1_ generation from a K326 background for TMV inoculation: a double mutant (7#), *NtTOM2A_T* single mutant (20#), and *NtTOM2A_S* single mutant (13#) (Fig. 5b, c and Supplementary Material l Fig. S7a, b, c, d). At 14 dpi, the double mutant 7# had no obvious mosaic phenotype compared to the wild type, whereas the two single mutants (13# and 20#) displayed phenotypes similar to that of the wild type (Fig. 5d). The disease grade of the wild type and the two single mutants were 9, while the 7# mutant was 1 (Fig. 5e). Moreover, western blot analysis showed that the accumulation of TMV in the upper leaves of the 7# mutant was considerably reduced compared with that in the wild type and the two single mutants (Fig. 5f; Supplementary Material 2).

### NtTOM2A allele distribution in tobacco germplasms

Using the variations identified in the *NtTOM2A* homeologs, we classified the allelic variations of the genes into three haplotypes: one associated with two simultaneous gene mutations (*NtTOM2A_*R), one associated with a *NtTOM2A_T* mutation (*NtTOM2A_*ST), and one associated with a *NtTOM2A_S* mutation (*NtTOM2A_*SS). Among the 577 accessions of tobacco germplasms (Supplementary Table S5), except for JT88, only four accessions belonging to *NtTOM2A_*R, 26 accessions belonging to *NtTOM2A_*ST, and one accession belonging to *NtTOM2A_*SS were identified (Table 1). These results suggested that NtTOM2A natural mutations were rarely selected for during tobacco breeding.

**Table 1:** Thirty-three tobacco germplasms and their mutations within *NtTOM2A_T* and *NtTOM2A_S.* NtTOM2A_T_M1: the mutation of *NtTOM2A_T*; NtTOM2A_S_M1: the SNP mutation of *NtTOM2A_S*; NtTOM2A_S_M2: the Indel mutation of *NtTOM2A_S;* -2 bp: two bases deletion; T → C: base mutation

Accession number	Germplasm name	NtTOM2A_T_M1	NtTOM2A_S_M1	NtTOM2A_S_M2
449	JT88	-2 bp	T → C	-2 bp
467	Liaoyan8	-2 bp		
1134	Ambalema	-2 bp	T → C	-2 bp
1187	No.888			-2 bp
1368	86–1	-2 bp		
1386	Anxuan3hao	-2 bp		
2266	K326			
2333	Huining1	-2 bp		
2453	CV16-2	-2 bp		
2480	taiyan8	-2 bp	T → C	-2 bp
2502	CV58	-2 bp		
2503	CV87	-2 bp		
2512	B13	-2 bp		
2514	B22	-2 bp		
3633	Xanthi Basma	-2 bp		
3658	Zhongyan99	-2 bp		
4411	98–15-2111	-2 bp		
4427	8021	-2 bp		
4763	CV89	-2 bp		
4779	Qinyan96	-2 bp		
4816	3116	-2 bp		
4824	Kang88	-2 bp		
4829	Zhongyan102	-2 bp		
4831	Longjiang981	-2 bp		
4851	Yunyan100	-2 bp		
5251	Taiyan10	-2 bp	T → C	-2 bp
5254	Taiyan11	-2 bp	T → C	-2 bp
5269	Yunyan105	-2 bp		
5272	Liaoyan9808	-2 bp		
5274	Yuyan10	-2 bp		
5290	CF87	-2 bp		
5293	CT107	-2 bp		
5382	9891	-2 bp		

**Fig. 6:**
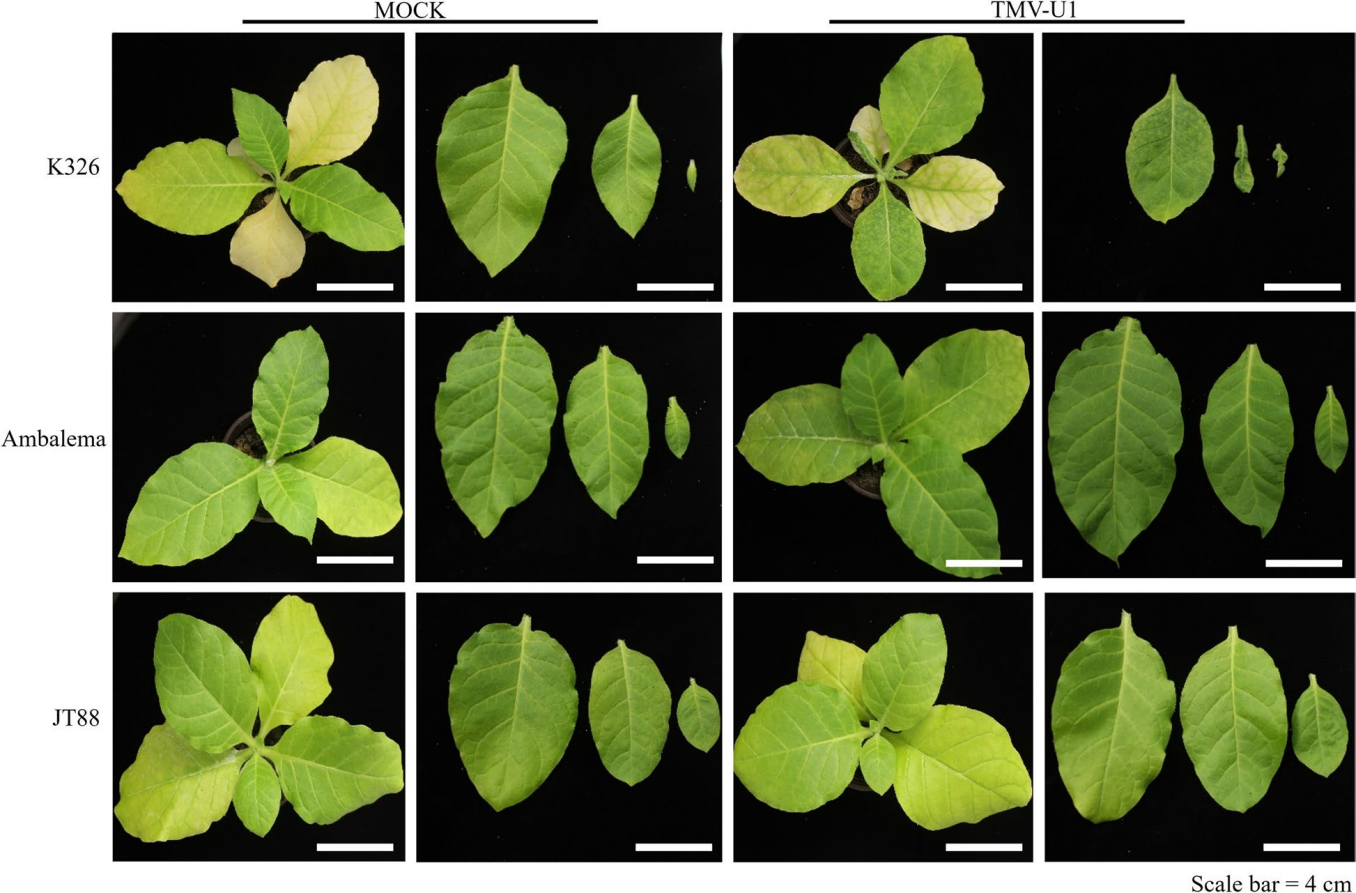
Phenotypic characterization of Ambalema upon TMV infection. The germplasm Ambalema harboring the same mutation as JT88 showed no obvious mosaic symptoms at 14 dpi. Scale bar = 4 cm

To further verify the role of NtTOM2A in TMV infection, we selected the accession Ambalema, which belongs to the *NtTOM2A_*R haplotype group, for TMV inoculation. After inoculation with TMV for 14 days, the mosaic phenotype was rarely observed in the upper leaves of Ambalema, similar to JT88 (Fig. 6). These results indicated that the simultaneous mutations of this two *NtTOM2A* homoeologs are responsible for TMV resistance.

## Discussion

### Genetic mapping of functional genes in tobacco

Forward genetics has long been difficult to apply to tobacco functional genomics because of inadequate genome assembly and highly repetitive sequences. Many functional genomic studies on tobacco have been conducted using reverse genetic approaches [48–50]. With the progress of studies on the tobacco reference genome and the development of high-throughput sequencing, the mapping of functional genes using forward genetics has become possible. Sequencing of the tobacco reference genome has enabled map-based cloning of homologous loci related to nitrogen utilization efficiency [51]. In the present study, we used the resistant germplasm JT88 and the susceptible cultivar K326 as genetic materials (Fig. 1a) and mapped *NtTOM2A_T* using BSA combined with map-based cloning in tobacco (Figs. 2c and 3a). A previous study showed that *NttTOM2A* (*TOM2A* of *Nicotiana tomentosiformis*) and *NtsTOM2A* (*TOM2A* of *Nicotiana sylvestris*) are responsible for TMV resistance in tobacco and tomato [39]. Hence, we aligned the NtsTOM2A sequence to the Yunyan87 reference genome and found that NtsTOM2A was located on Contig8852. Our BSA did not detected NtsTOM2A as the sequencing depth was not enough. Subsequently, the homeolog of *NtTOM2A_T*, designated as *NtTOM2A_S*, was examined, revealing an SNP in the fifth exon and a 2 bp deletion in the sixth exon of JT88 (Fig. 4a). Thus, we showed that *NtTOM2A_T* and *NtTOM2A_S* are candidate homeologs for TMV resistance in JT88.

### Wild TOM2As are required for TMV replication

The replication of plant viruses depends on host factors. *Tobamovirus* multiplication proteins are specialized host factors related to *Tobamovirus* replication in *A. thaliana*, tobacco, and tomato [4, 34–36]. *AtTOM2A*, *AtTOM1*, and *AtTOM3* are members of the TMV replication complex in *Arabidopsis* [38]. A previous study demonstrated that in *A. thaliana*, tobamoviruses require both TOM1 and TOM2A to build a replication complex during the early stages of infection; however, in later stages of infection, the replication complex is also formed without TOM2A [52]. In tobacco, simultaneous silencing of *NtTOM1* and *NtTOM3* almost completely inhibits the replication of TMV [33]. Moreover, simultaneous loss-of-function mutations in *NtTOM2As* lead to an asymptomatic phenotype [39]. In the present study, the simultaneous mutation of two homeologs, namely *NtTOM2A_T* and *NtTOM2A_S*, suppressed the accumulation of TMV in K326, while mutations in either *NtTOM2A_T* or *NtTOM2A_S* alone did not suppress TMV replication (Fig. 5d, e, f). These results demonstrated that, as integral membrane proteins, *NtTOM2A* and *NtTOM1/NtTOM3* are necessary for TMV replication [33, 39]. However, the roles of NtTOM2As in the replication complex require further study.

### TOM2A may affect plant growth and development

TOM2A is a member of the tetraspanin (TM4SF) family and contains a tetraspanin domain (PF00335). Tetraspanin family members are widely distributed in animals and plants, from lower eukaryotic yeasts to humans, and play important roles in reproduction, growth, and disease resistance [53–55]. In animals, tetraspanin proteins are favorable for multiple viral infections. When their expression is repressed, hosts become more resistant to viruses such as human papillomavirus (HPV) and feline immunodeficiency virus (FIV) [56, 57]. However, in plants, the research on *tetraspanin* genes is very limited. In *A. thaliana*, the gene family plays a crucial role in plant growth and development. For example, *tetraspanin1/tornado2/ekeko* participates in leaf and root patterning, while *tetraspanin3* functions in cell-to-cell communication during plant development [58, 59]. *AtAAF* regulates flower organ size and affects various developmental processes [60]. Comprehensive expression profiling of rice *tetraspanin* genes has revealed their diverse roles in plant development and response to abiotic stress [61]. In *Arabidopsis*, a triple mutant deficient in all three *AtTOM2A* homologs grew more slowly than the wild type [38]. In tomato, mutants with null alleles of *SlTOM2A* showed bent vegetative and floral branches. In our study, the CRISPR/Cas9 line *nttom2a* showed shortened plant height, but have no obvious influence on leaf length and width (Supplementary Material l Fig. S8a, b, c, d, e). Thus, to better exploit *NtTOM2As* in TMV resistance breeding, their functions in tobacco growth and development should be further studied.

Among the germplasms identified in the present study, only flue-cured tobacco taiyan8, taiyan10, taiyan11, and sun-cured tobacco Ambalema possessed a mutation within both *NtTOM2A_T* and *NtTOM2A_S* (Table 1). Germplasms harboring the *NtTOM2A_*R haplotype did not show dwarfing or bent flower stem phenotype (data not shown). However, the *Arabidopsis* and tomato mutants with full loss-of-function of the TOM2A homologs displayed abnormal phenotypes. The natural variations in *NtTOM2As* in JT88 resulted in a truncated NtTOM2A_T protein, but the predicted protein structure of NtTOM2A_S changed slightly (Figs. 3b, 4b). The relatively intact NtTOM2A_S protein might contribute to the function of NtTOM2A_S normal growth in the resistant germplasms. Previous studies have shown that TOM2A interacts with TOM1 to promote TMV replication, but the crucial interaction region has not yet been determined. Identification of the interaction region and precise editing would be an ideal strategy for obtaining TMV resistant tobacco cultivars without growth penalties.

## Conclusion

In this study, we identified a tobacco germplasm, JT88, a new TMV resistant cultivar with high resistance to TMV, and mapped two homeologs responsible for TMV replication. Our findings provide evidence that the simultaneous mutation of two *NtTOM2A* homeologs confers TMV resistance in tobacco. Our results also reveal the rarity of the *NtTOM2A_*R haplotype in tobacco germplasms and its potential value in TMV resistance breeding. Further studies are needed to investigate the potential functions of NtTOM2A in plant growth and development.

## Materials and methods

### Plant materials and mapping population construction

Seeds of the tobacco accessions used in this study were provided by the National Tobacco Germplasm Resource Medium-term Bank (Qingdao, China). The TMV-susceptible cultivar *N. tabacum* cv. K326 (maternal donor) (Accession ID:2266) was crossed with the resistant line *N. tabacum* cv. JT88 (paternal donor) (Accession ID:449) to generate the first filial generation (F_1_), which was then self-crossed to produce F_2_ generation plants for resistance identification and BSA. All plants were grown in an environmental chamber with a photoperiod of 16 h light/8 h dark at 26℃, and the relative humidity was 70% ± 5%.

### Virus and virus inoculation

The tobacco mosaic virus strain TMV-U1 was propagated and maintained by the Tobacco Research Institute of the Chinese Academy of Agricultural Sciences. Virus inoculation was performed according to Höller’s method with slight modifications [62]. Six-week-old seedlings were used for virus inoculation, and each plant was inoculated with two leaves. Every 0.1 g TMV-infected leaf was homogenized in 4 ml phosphate buffer saline (PBS) and used swabs to inoculate the healthy leaves which spread silica sand on the surface.

### Phenotype scoring and BSA-Seq analysis

Fourteen days after the inoculation with TMV-U1, disease grade (dg) was scaled to 0, 1, 3, 5, 7, and 9 as previously described (Supplementary Material l Fig. S2) [63, 64]. Twenty F_2_ individuals with extreme resistance (dg = 0) were assigned to the “resistant bulk” (R bulk) group and 20 F_2_ individuals with extreme susceptibility (dg = 9) were assigned to the “susceptible bulk” (S bulk) group. In addition, F_2:3_ populations derived from the 20 F_2_ resistant individuals were used to confirm the TMV resistance of the corresponding F_2_ recombinants for BSA. The mean disease index of the infected tobacco plants was calculated according to previous method [63, 64].

The Deoxyribonucleic acid (DNA) of K326, JT88, and the F_2_ individuals was extracted from young leaves using the CTAB method [65]. The qualified DNA samples were randomly broken into fragments of 350 bp using a Covaris ultrasonicator. The Illumina TruSeq library construction kit was used to construct the library, which was sequenced using an Illumina Hiseq™ PE150 platform (Beijing Novogene Bioinformatics Institute Co., Ltd, Beijing, China). Clean reads from the bulk DNA were aligned with the *N. tabacum* cv. Yunyan87 reference genome (kindly offered by Dr. He Xie) using the Burrows–Wheeler Aligner software [66]. The Samtools software [67] was used to remove duplicates from the comparison results. The UnifiedGenotyper module of the GATK3.8 software [68] was used to detect all SNPs, after which each SNP was annotated using ANNOVAR software [69]. To determine the distribution of the offspring SNP index on the chromosomes, a sliding window analysis with a 1-Mb window size and 1-Kb increment was used to calculate the average SNP-index of the SNPs. The ΔSNP-index was calculated using the following formula:

$$\Delta {\text{SNP}}-{\text{index}}={\text{SNP}}-\mathrm{index }(\mathrm{bulk B}) -{\text{SNP}}-\mathrm{index }(\mathrm{bulk A})$$

The 95% confidence level was selected as the screening threshold, and the permutation test was repeated 1000 times to generate the confidence intervals.

### Marker development and fine genetic mapping

DNA templates of K326, JT88, F_1_, R bulk, and S bulk were utilized to screen for polymorphic markers by using published SSR markers [46]. To increase the density of genetic markers, SNP markers were designed based on the sequence differences between K326 and JT88, and a skeleton physical map was constructed using genome-wide polymorphic SNP and SSR markers. Eventually, six markers and 1440 F_2_ individuals were used for genetic mapping. The crossover rate was used to calculate the genetic distances between the candidate genes with markers. All primers used for genetic mapping are listed in Supplementary Table S6.

### Identification of candidate genes

The DNA of *N. tabacum* cv. K326 and *N. tabacum* cv. JT88 was used as an amplification template. Polymerase chain reaction (PCR) was performed using a high-fidelity thermostable DNA polymerase (P525, Vazyme, China), and the PCR products were sequenced by Sanger sequencing (Ruibo, China). Sequence alignment was performed using the SnapGene® 5.2 software. The gene structure was drawn using the GSDS2.0 online software [70] (http://gsds.gao-lab.org/), and deduced three-dimensional structures of the protein were predicted using online SWISS-MODEL software [71] (https://swissmodel.expasy.org/).

### Plasmid construction and tobacco transformation

The gene-editing vector pkse401 was obtained from Qijun Chen (Addgene plasmid # 62202) [72], and *pkse401-NtTOM2A* was constructed to edit *NtTOM2A_T* and *NtTOM2A_S* using CRISPR/Cas9. sgRNA targeting a region within exon 1 of *NtTOM2A_T* and *NtTOM2A_S* were designed using the online tool CRISPR MultiTargeter [47]. Tobacco transformation was performed using the leaf disc method as described previously [73]. The T_1_ generation of knockout transgenic lines was used for viral inoculation analysis. All primers used for vector construction and targeted mutant identification are listed in Supplementary Table S6.

### Western blot analysis

Total proteins for the WB assay were extracted from the leaf tissues using a Thermo Scientific Protein Extraction Kit (#78,835, Thermo Scientific, USA) according to the manufacturer’s instructions. The TMV CP was detected using a specific antibody against TMV CP (Agdia, USA), and β-actin was used as an internal reference. WB was performed as described [74].

### Gene expression analysis

Tobacco apical non-inoculated leaves were harvested at 12 dpi with TMV for Ribonucleic acid (RNA) extraction and quantitative reverse transcription polymerase chain reaction (qRT-PCR). Total RNA was isolated using the Plant Total RNA Kit ZP405 (Beijing Zoman Biotechnology Co., Ltd., China) according to the manufacturer’s instructions. Reverse transcription was performed using a HiScript III 1st Strand cDNA Synthesis Kit (Vazyme, China). The expression level of the TMV coat protein gene was normalized to that of the tobacco *actin* gene and quantified using the 2^−∆∆CT^ method [75]. Error bars represent the standard deviations (SDs) of three biological replicates. All primers used for the gene expression analysis are listed in Supplementary Table S6.

### Phylogenetic analysis, sequence alignment, and three-dimensional (3d) folding structure prediction

The amino acid sequences of NtTOM2A_T, NtTOM2A_S, NtomTOM2A, and NsylTOM2A were used to investigate the phylogenetic relationships. Sequences were aligned using SnapGene® 5.2 software, and the alignments were visualized in GeneDoc software. Three-dimensional (3d) folding structure prediction was performed using SWISS-MODEL (https://swissmodel.expasy.org/) [71].

### Allelic variation analysis of NtTOM2A in tobacco germplasms

For the allelic variation analysis of *NtTOM2A*, three mutation sites (two INDELs and one SNP) in *NtTOM2A_T* and *NtTOM2A_S* were used to access the haplotypes of *NtTOM2A*. The DNA templates of 577 tobacco germplasms (*N. tabacum*) were amplified with the primers listed in Supplementary Table S6 using a 2 × Taq Master Mix (P222, Vazyme, China), and the PCR products were sequenced by Sanger sequencing (Ruibo, China). The SnapGene® 5.2 software was used for sequence alignment.

### Statistical analysis

Statistical analyses were performed using GraphPad Prism Version 8.0.2 software (GraphPad Software, San Diego, California USA). Experimental data were analyzed using Student’s *t*-test. ****:*P* < 0.0001.

### Complies with international, national, and/or institutional guidelines

Experimental research and field studies on plants (either cultivated or wild) comply with relevant institutional, national, and international guidelines and legislation.

### Supplementary Information

**Additional file 1:** **Figure S1. ***N* gene detection by tobacco mosaic virus (TMV) inoculation and *N*-maker amplification. **Figure S2.** Plant phenotype of F_2_ populations at 14 dpi. **Figure S3. **Genetic mapping of the *NtTOM2A_T* homeolog by bulked segregant analysis and map-based cloning. **Figure S4. **Phylogenetic analysis and sequence alignment of *NtTOM2A_T*, *NtTOM2A_S*, *NtomTOM2A*, and *NsylTOM2A* alleles. **Figure S5. **Sequence alignment of the *NtTOM2A_T* coding sequence and amino acid sequence between K326 and JT88. **Figure S6. **Sequence alignment of the *NtTOM2A_S* coding sequence and amino acid sequence between K326 and JT88. **Figure S7.** Sequence alignment of NtTOM2A_T and NtTOM2A_S coding sequence and amino acid sequence between the wild type (WT) and the three mutants. **Figure S8.** Phenotype of wild type and *ntttom2a* mutant. 

**Additional file 2: Supplementary Figure 1d.** The original figure of WB analysis (IL). The purpose bands were highlighted in red frame. **Supplementary Figure 1d.** The original figure of WB analysis (AL). The purpose bands were highlighted in red frame. **Supplementary Figure 5e.** The original figure of WB analysis. The purpose bands were highlighted in red line. **Supplementary Figure S1a.** The full-length gel image of Fig S1a. The purpose bands were highlighted in red frame. **Supplementary Figure S3c.** The full-length gel image of Fig S3c. The purpose bands were highlighted in red frame. 

**Additional file 3:  Supplementary Table 1.** Sequencing information of four samples. 

**Additional file 4:  Supplementary Table 2.** SNP numbers of each chromosomes and scaffolds.

**Additional file 5:  Supplementary Table 3.** SNP makers detected in this study.     

**Additional file 6:  Supplementary Table 4.**  The predicted genes in the candidate interval.

**Additional file 7:  Supplementary Table 5.** Tobacco germplasms used in this study and corresponding accession numbers. 

**Additional file 8:  Supplementary Table 6.** Sequences of primers used in this study. 

## Data Availability

The whole genome resequencing information of two bulks (PRJCA015061) can be found online at National Genomics Data Center (https://ngdc.cncb.ac.cn/).
