# Supplementary material for: Fine mapping and identification of two NtTOM2A homeologs responsible for tobacco mosaic virus replication in tobacco (Nicotiana tabacum L.)
Source: BMC Plant Biol. 2024 Jan 24;24:67. doi: 10.1186/s12870-024-04744-y (PMC10807211; doi:10.1186/s12870-024-04744-y)
Supplement: Supplementary file 1 — Additional file 1: Figure S1. N gene detection by tobacco mosaic virus (TMV) inoculation and N-maker amplification. Figure S2. Plant phenotype of F2 populations at 14 dpi. Figure S3. Genetic mapping of the NtTOM2A_T homeolog by bulked segregant analysis and map-based cloning. Figure S4. Phylogenetic analysis and sequence alignment of NtTOM2A_T, NtTOM2A_S, NtomTOM2A, and NsylTOM2A alleles. Figure S5. Sequence alignment of the NtTOM2A_T coding sequence and amino acid sequence between K326 and JT88. Figure S6. Sequence alignment of the NtTOM2A_S coding sequence and amino acid sequence between K326 and JT88. Figure S7. Sequence alignment of NtTOM2A_T and NtTOM2A_S coding sequence and amino acid sequence between the wild type (WT) and the three mutants. Figure S8. Phenotype of wild type and ntttom2a mutant. [file 12870_2024_4744_MOESM1_ESM.docx]

Supplementary Material

**Fine mapping and identification of two *NtTOM2A* homeologs responsible for tobacco mosaic virus replication in tobacco** **(*****Nicotiana tabacum* L.)**

Xuebo Wang^1,2†^, Zhan Shen^1†^, Caiyue Li^1^, Yalin Bai^1^, Yangyang Li^3^, Wenhui Zhang^4,5^, Zunqiang Li^6*^,Caihong Jiang^1^, Lirui Cheng^1^, Aiguo Yang^1*^, Dan Liu^1*^

^1^ Key Laboratory for Tobacco Gene Resources, Tobacco Research Institute, Chinese Academy of Agricultural Sciences (CAAS), Qingdao, 266101, China

^2^ Tobacco Science Research Institute of Guangdong Province, Shaoguan 512029, Guangdong, China

^3^ Hunan Tobacco Research Institute, Changsha, 410004, China

^4^ Linyi University, Linyi, 276000, Shandong, China

^5^ Philippine Christian University Center for International Education, Manila, 1004, Philippine

^6^ Tobacco Research Institute of Mudanjiang, Harbin, 150076, China

*** Correspondence:**

Zunqiang Li; Email: lizunqiangq@163.com

Aiguo Yang; Email: [yangaiguo@caas.cn](mailto:yangaiguo@caas.cn)

Dan Liu; Email: [liudan@caas.cn](mailto:liudan@caas.cn)

Xuebo Wang and Zhan Shen contributed equally to this work

**
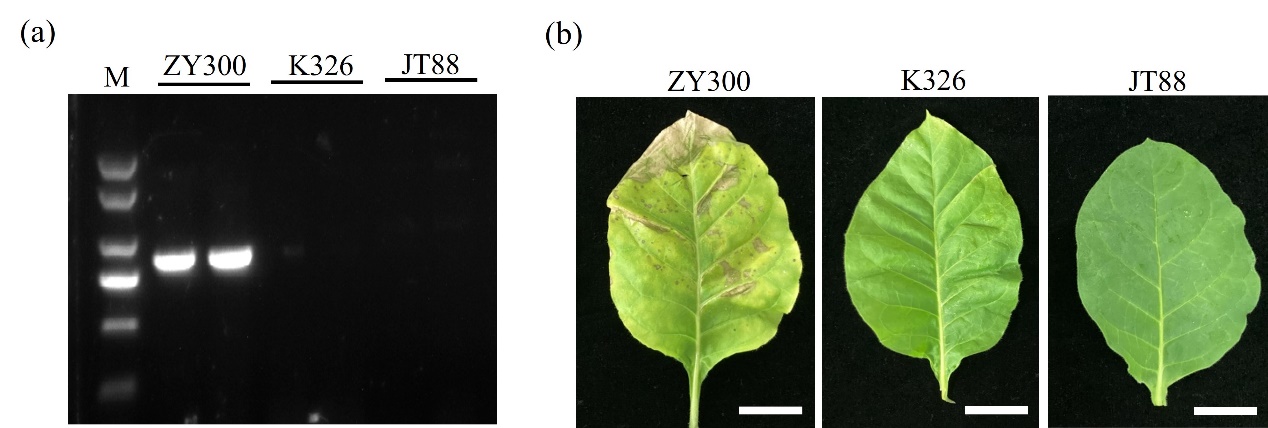
**

**Figure S1** *N* gene detection by tobacco mosaic virus (TMV) inoculation and *N*-maker amplification. **(a)** *N*-maker amplification of the three varieties by polymerase chain reaction (PCR). M: DL2000. **(b)** The hypersensitive reaction (HR) phenotype of the three varieties at 7 days post inoculation with TMV .Scale bar = 3 cm.

**
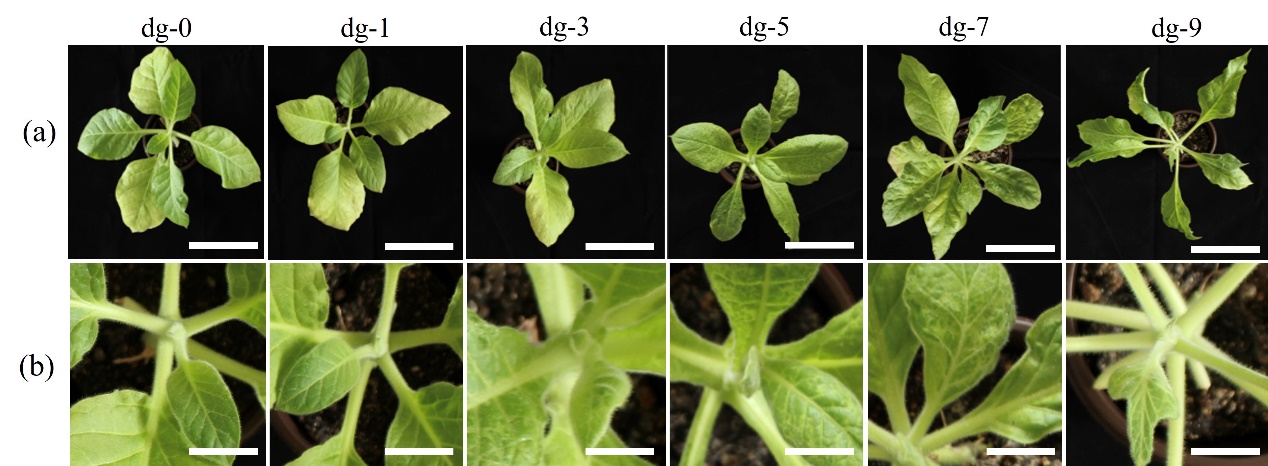
**

**Figure S2** Plant phenotype of F_2_ populations at 14 dpi. **(b)** is the local zoom of **(a)**. Disease grade was determined by visual scoring according to GB/T 23222-2008, and the number represents disease severity. dg: disease grade. Scale bars = 12 cm (a), and 4 cm (b).


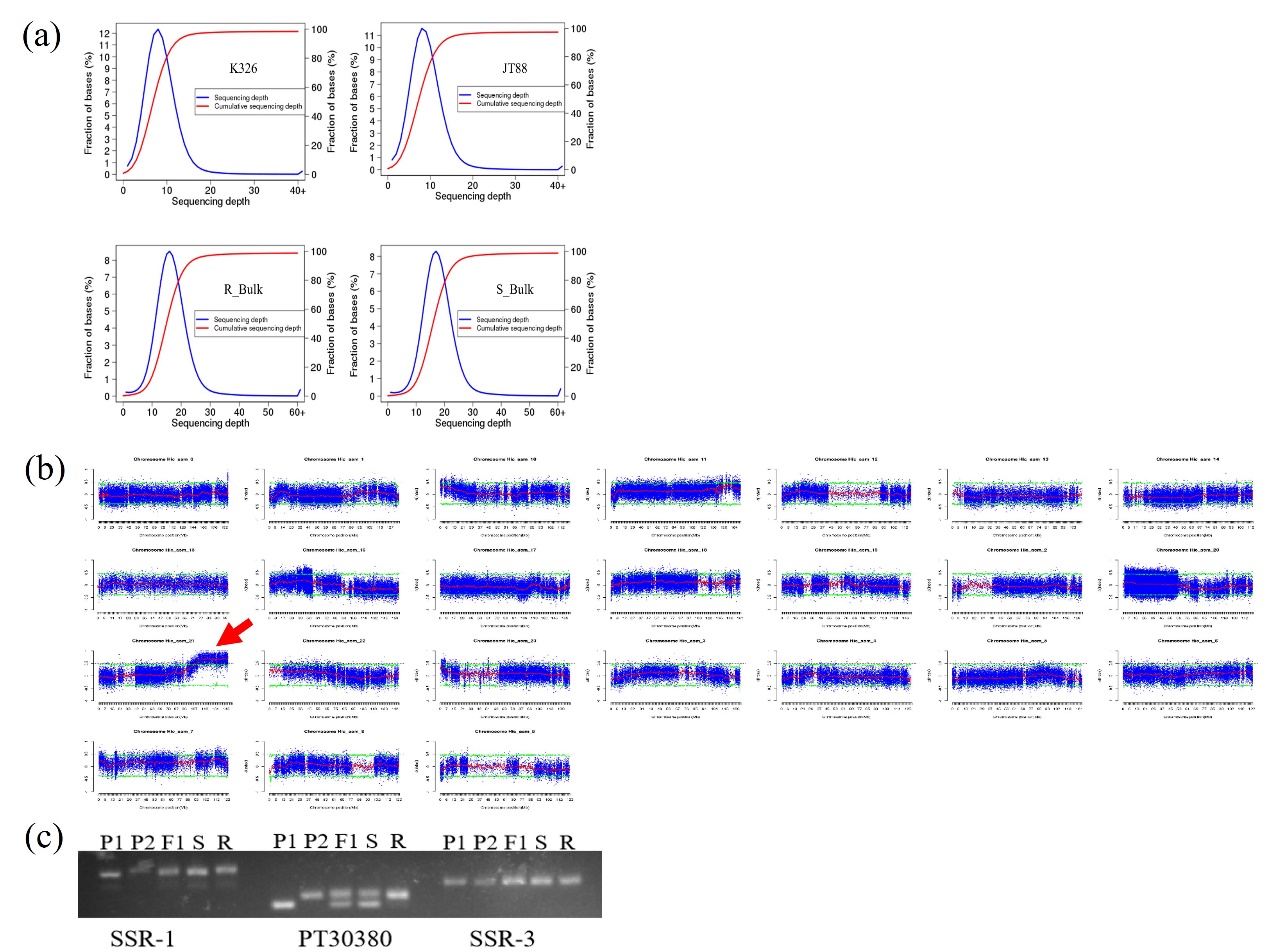


**Figure S3** Genetic mapping of the *NtTOM2A_T* homeolog by bulked segregant analysis and map-based cloning. **(a)** Sequencing depth of K326, JT88, S_bulk, and R_bulk. The abscissa represents the sequencing depth, the blue line and left ordinate axis in the figure represent the percentage of the corresponding depth in the whole genome, and the red line and right ordinate in the figure represent the percentage of the sites less than or equal to the depth in the whole genome. **(b)** Distribution of the $\Delta$_all-index of two progenies in the partite chromosome. **(c)** Polymorphic simple sequence repeat (SSR) marker screening. P1: K326, P2: JT88, F1: K326×JT88 F_1_ population, S: S_bulk, R: R_bulk.

**
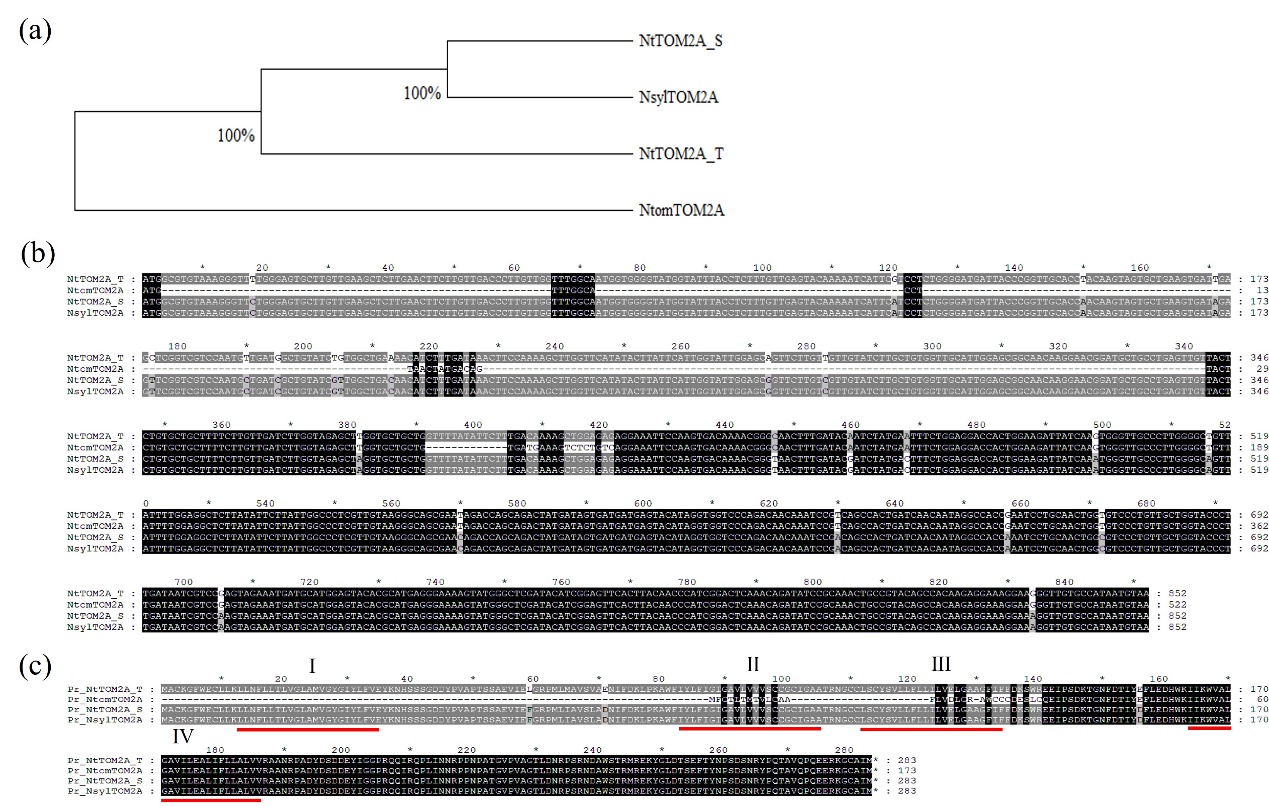
**

**Figure S4** Phylogenetic analysis and sequence alignment of *NtTOM2A_T*, *NtTOM2A_S*, *NtomTOM2A*, and *NsylTOM2A* alleles. **(a)** Phylogenetic analysis of *NtTOM2A_T*, *NtTOM2A_S*, *NtomTOM2A*, and *NsylTOM2A*. **(b)** Sequence alignment of *TOM2A* coding sequence between *Nicotiana tabacum*, *N. sylvestris*, and *N. tomentosiformis*. **(c)** Deduced TOM2A amino acid sequence alignment between *N. tabacum*, *N. sylvestris*, and *N. tomentosiformis*.

**
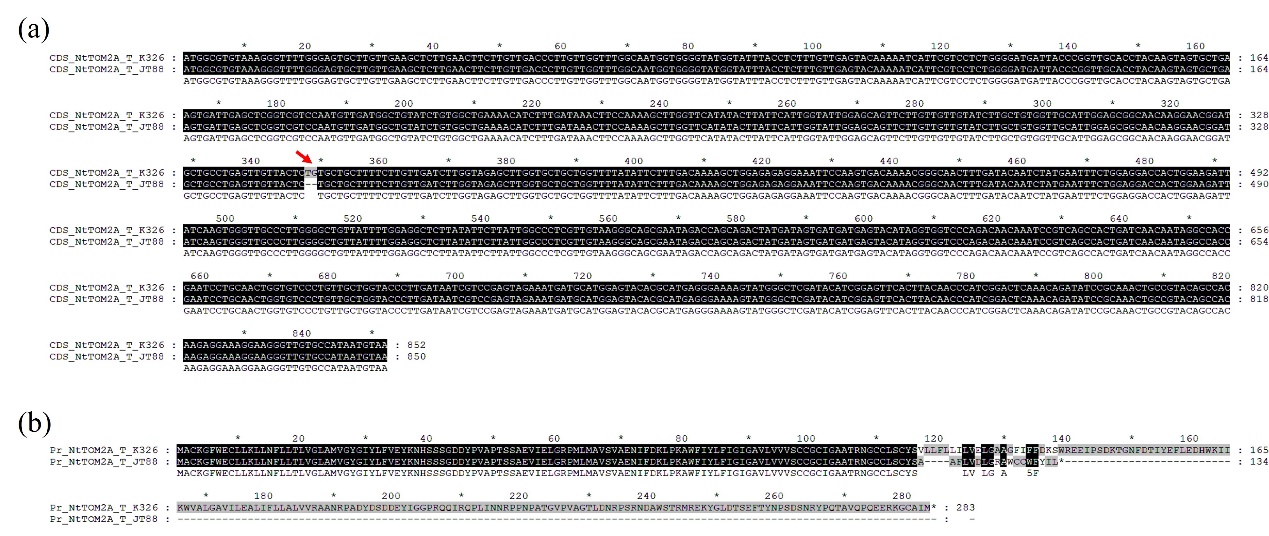
**

**Figure S5** Sequence alignment of the *NtTOM2A_T* coding sequence and amino acid sequence between K326 and JT88. **(a)** Coding sequence alignment. **(b)** Deduced amino acid sequence alignment. The mutation sites were highlighted in red arrow.

**
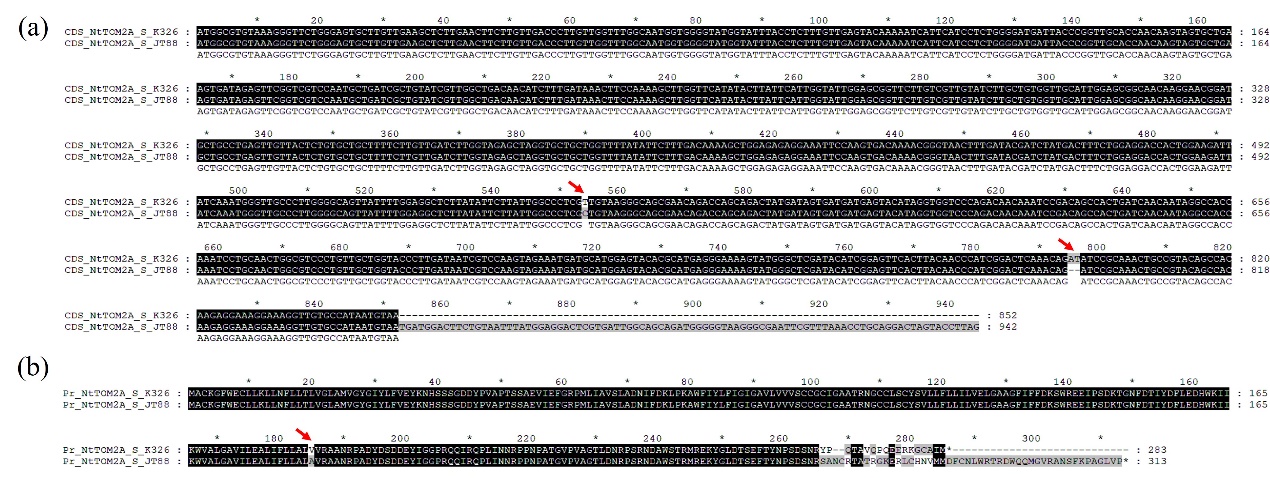
**

**Figure S6** Sequence alignment of the *NtTOM2A_S* coding sequence and amino acid sequence between K326 and JT88. **(a)** Coding sequence alignment. **(b)** Deduced amino acid sequence alignment. The mutation sites were highlighted in red arrow.

**
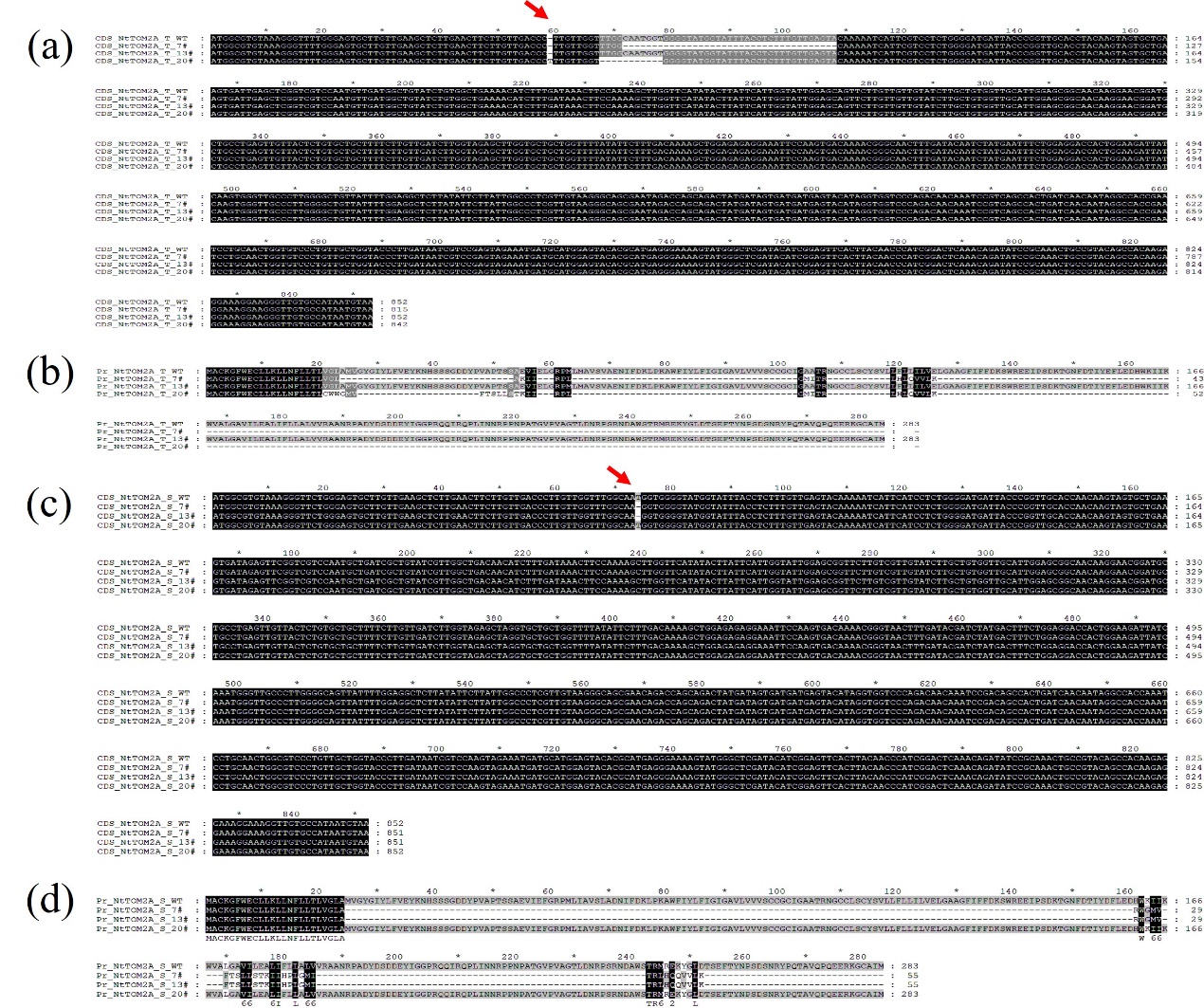
**

**Figure S7** Sequence alignment of NtTOM2A_T and NtTOM2A_S coding sequence and amino acid sequence between the wild type (WT) and the three mutants. **(a)** Coding sequence alignment of the *NtTOM2A_T* genes. **(b)** Deduced amino acid sequence alignment of NtTOM2A_T. **(c)** Coding sequence alignment of the *NtTOM2A_S* gene. **(d)** Deduced amino acid sequence alignment of NtTOM2A_S. The mutation sites were highlighted in red arrow.


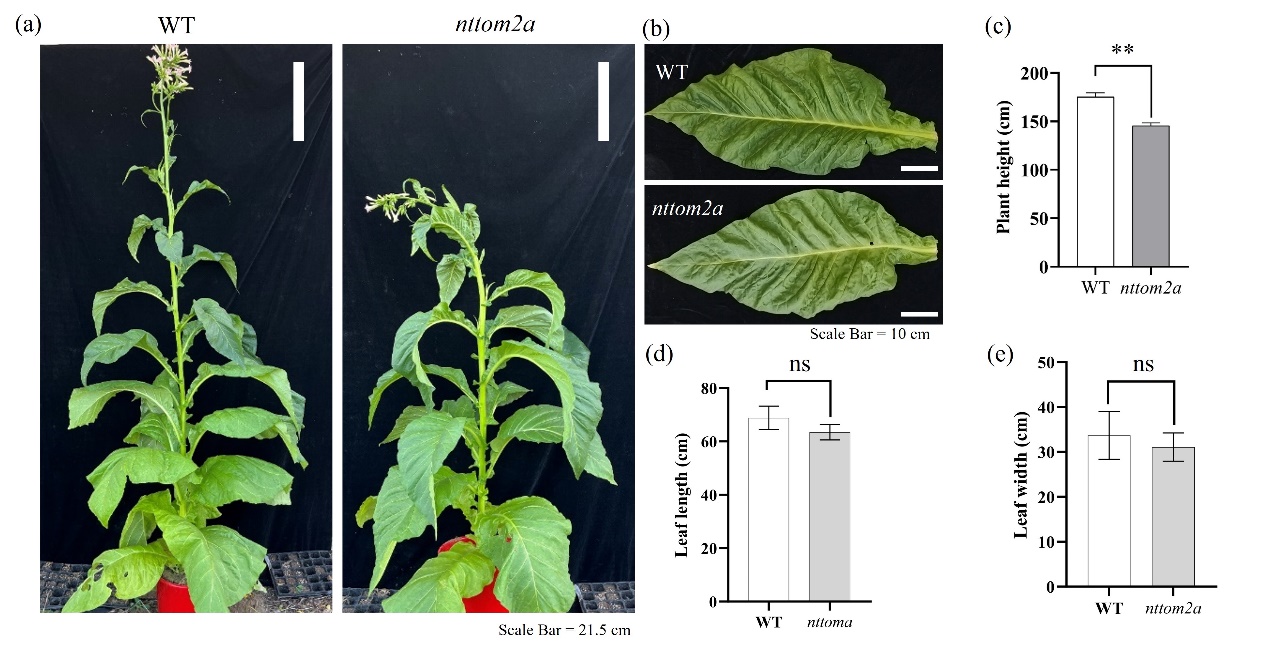


**Figure S8** Phenotype of wild type and *ntttom2a* mutant. **(a)** The overall plant phenotype of wild type and *ntttom2a* mutant. Scale Bar = 21.5 cm. **(b)** Leaf phenotype of wild type and *ntttom2a* mutant. Scale Bar = 10 cm. **(c)** Plant height of wild type and *ntttom2a* mutant. **(d)** Leaf length of wild type and *ntttom2a* mutant. **(e)** Leaf width of wild type and *ntttom2a* mutant. Student t-tests were used to determine the significance of differences between WT and *nttom2a* mutant (***P*＜0.01; ns: no significance).

**Supplementary Table legends**

Supplementary Table 1 Sequencing information of four samples

Supplementary Table 2 SNP numbers of each chromosome and scaffolds

Supplementary Table 3 SNP makers detected in this study

Supplementary Table 4 The predicted genes in the candidate interval

Supplementary Table 5 Tobacco germplasms used in this study and corresponding accession numbers

Supplementary Table 6 Sequences of primers used in this study
