# Supplementary material for: Fine mapping and identification of two NtTOM2A homeologs responsible for tobacco mosaic virus replication in tobacco (Nicotiana tabacum L.)
Source: BMC Plant Biol. 2024 Jan 24;24:67. doi: 10.1186/s12870-024-04744-y (PMC10807211; doi:10.1186/s12870-024-04744-y)
Supplement: Supplementary file 2 — Additional file 2: Supplementary Figure 1d. The original figure of WB analysis (IL). The purpose bands were highlighted in red frame. Supplementary Figure 1d. The original figure of WB analysis (AL). The purpose bands were highlighted in red frame. Supplementary Figure 5e. The original figure of WB analysis. The purpose bands were highlighted in red line. Supplementary Figure S1a. The full-length gel image of Fig S1a. The purpose bands were highlighted in red frame. Supplementary Figure S3c. The full-length gel image of Fig S3c. The purpose bands were highlighted in red frame. [file 12870_2024_4744_MOESM2_ESM.pptx]

## Slide 1
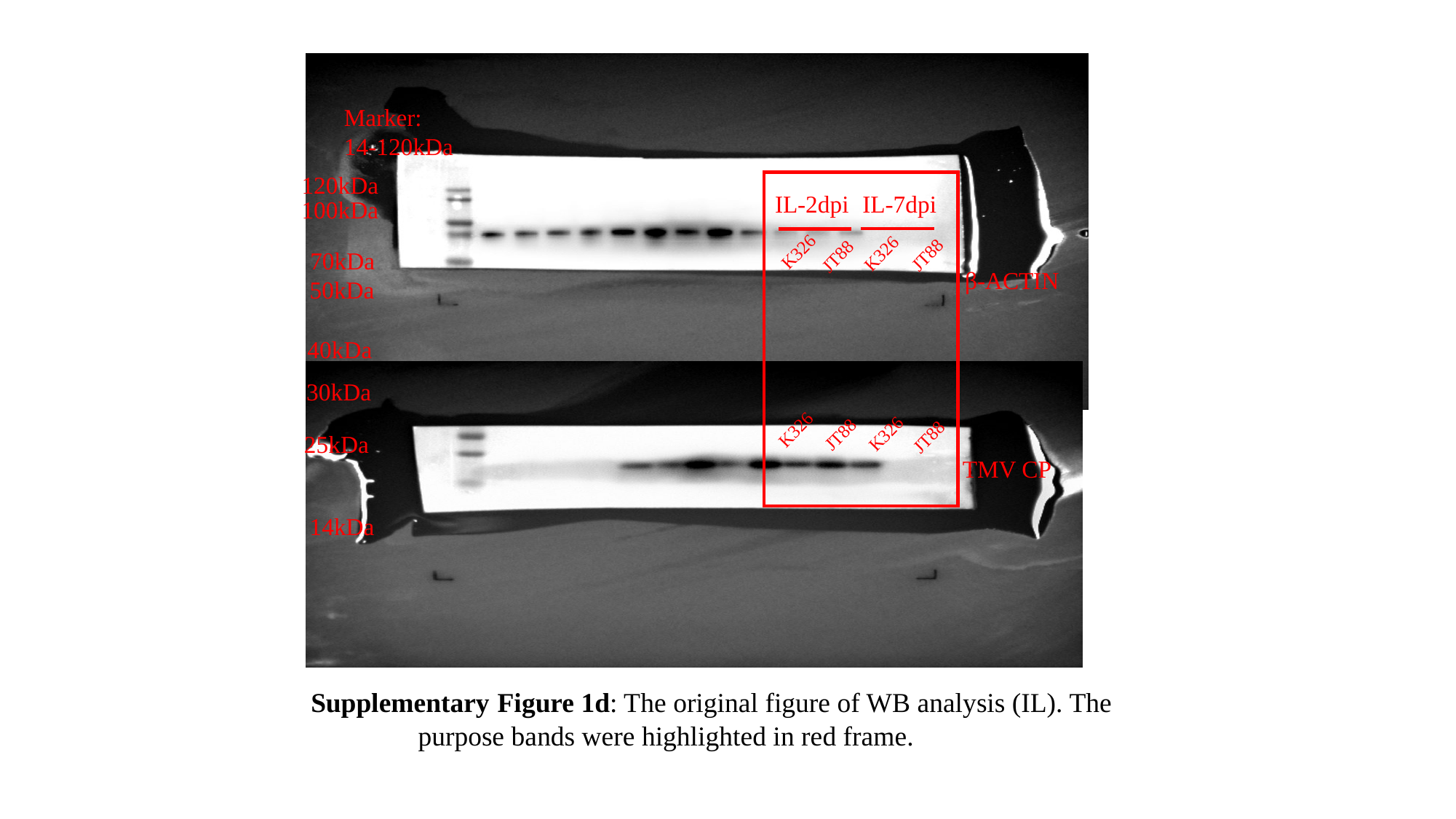

Marker:
14-120kDa
120kDa
IL-2dpi
IL-7dpi
100kDa
K326
JT88
K326
JT88
70kDa
β-ACTIN
50kDa
40kDa
30kDa
K326
JT88
K326
JT88
25kDa
TMV CP
14kDa
Supplementary Figure 1d: The original figure of WB analysis (IL). The purpose bands were highlighted in red frame.

## Slide 2
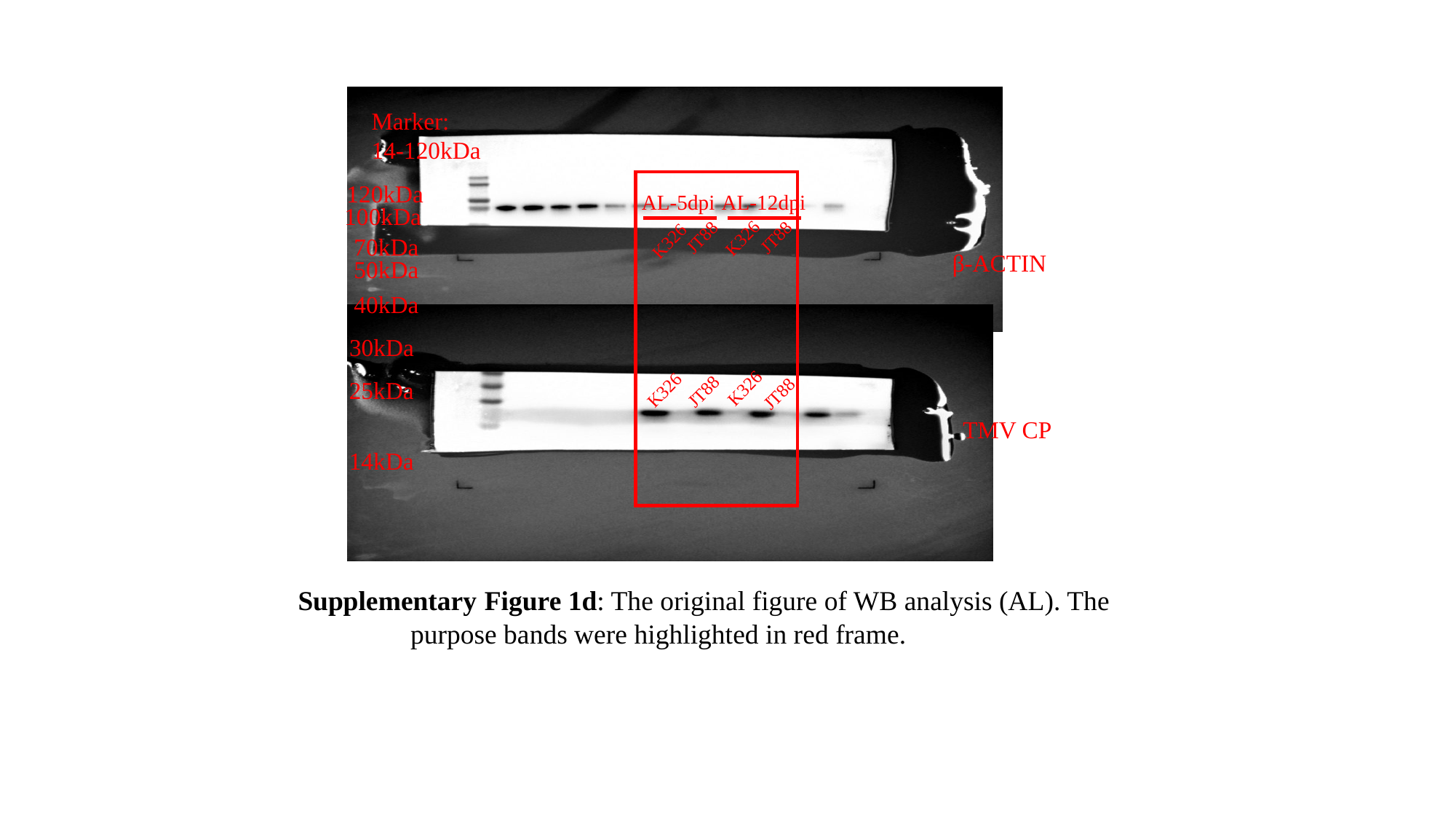

Marker:
14-120kDa
120kDa
AL-12dpi
AL-5dpi
100kDa
JT88
JT88
K326
K326
70kDa
β-ACTIN
50kDa
40kDa
30kDa
K326
JT88
K326
JT88
25kDa
TMV CP
14kDa
Supplementary Figure 1d: The original figure of WB analysis (AL). The purpose bands were highlighted in red frame.

## Slide 3
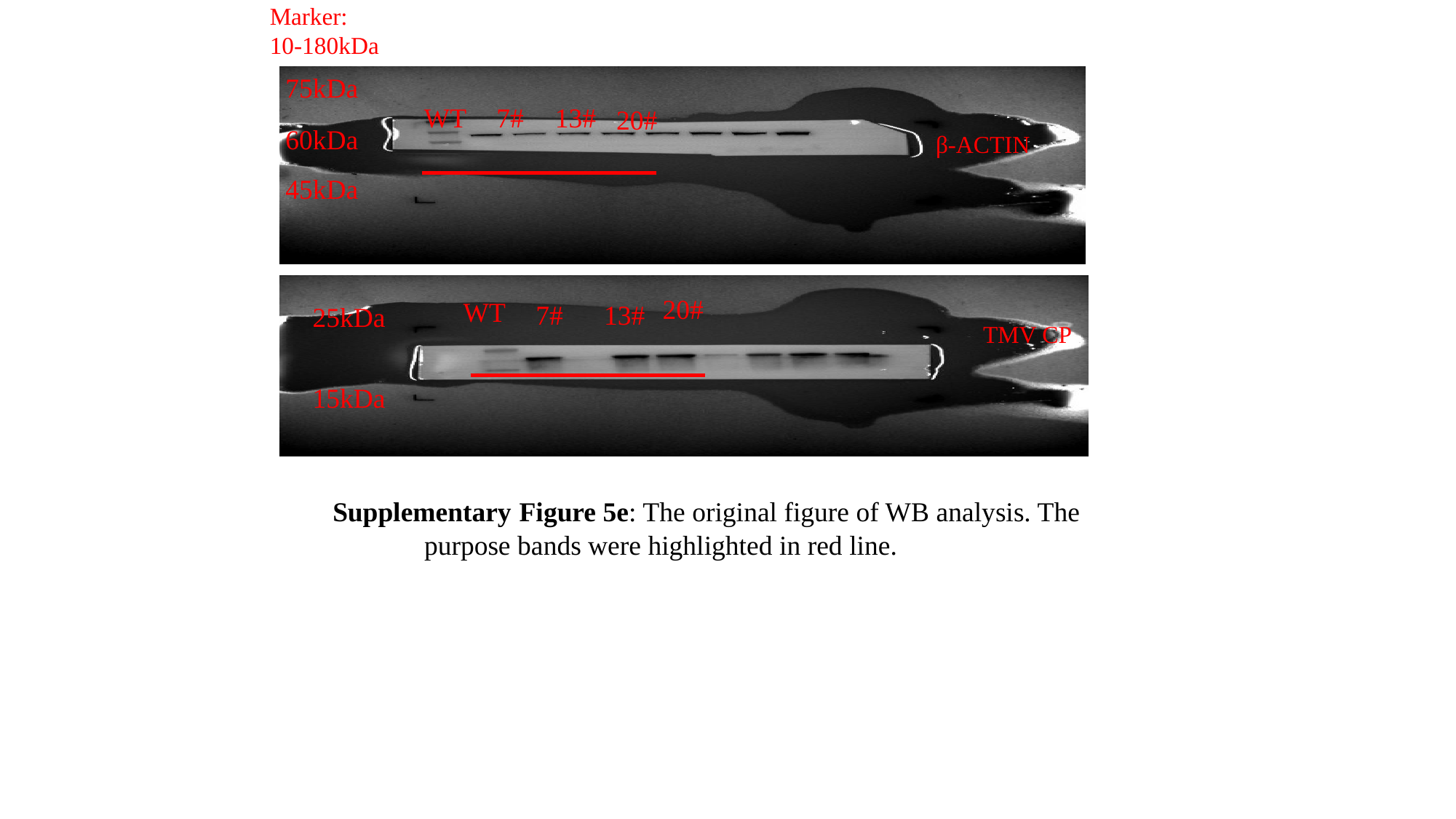

Marker:
10-180kDa
75kDa
WT
7#
13#
20#
60kDa
β-ACTIN
45kDa
20#
WT
7#
13#
25kDa
TMV CP
15kDa
Supplementary Figure 5e: The original figure of WB analysis. The purpose bands were highlighted in red line.

## Slide 4
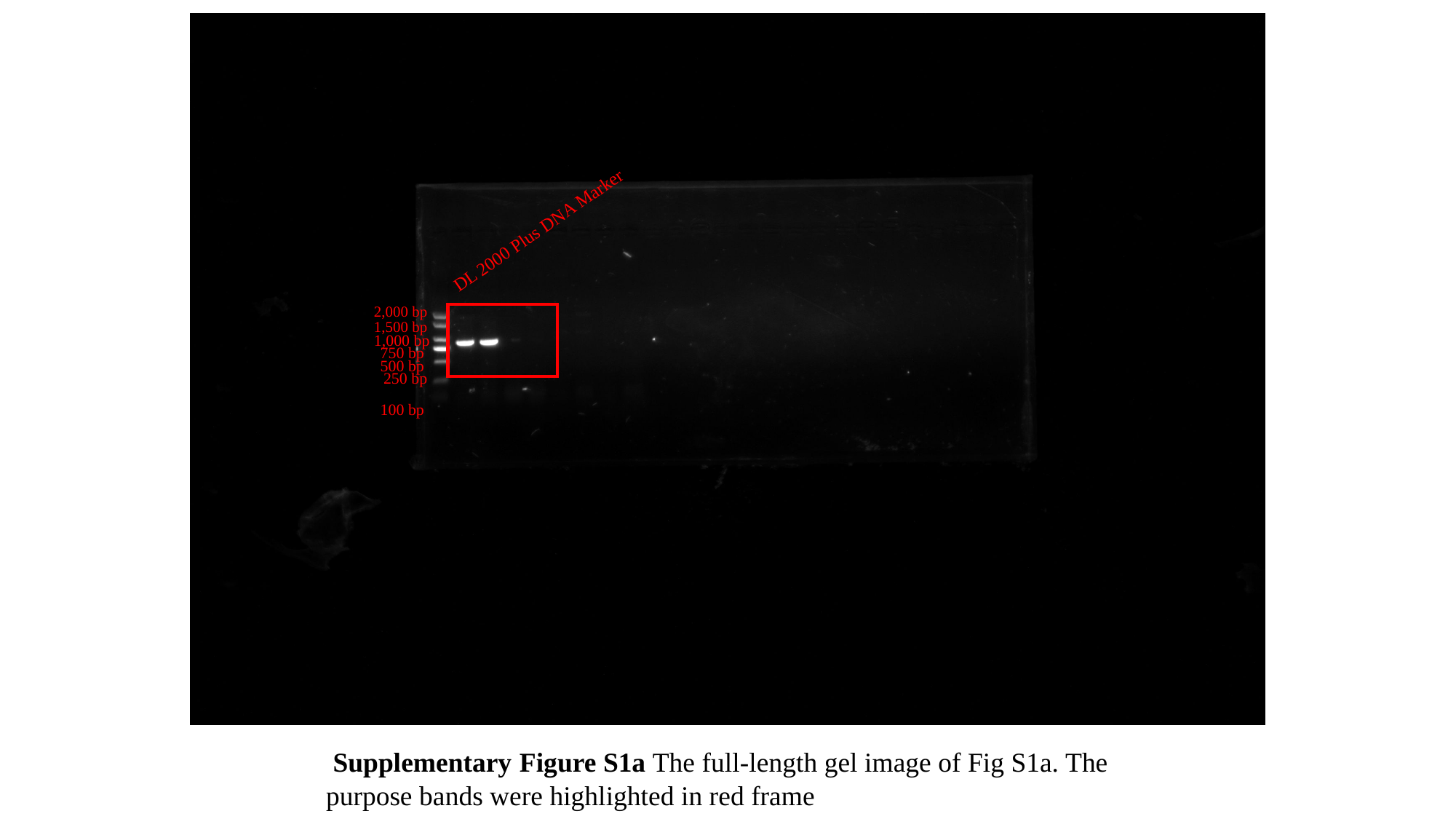

DL 2000 Plus DNA Marker
2,000 bp
1,500 bp
1,000 bp
750 bp
500 bp
250 bp
100 bp
 Supplementary Figure S1a The full-length gel image of Fig S1a. The purpose bands were highlighted in red frame

## Slide 5
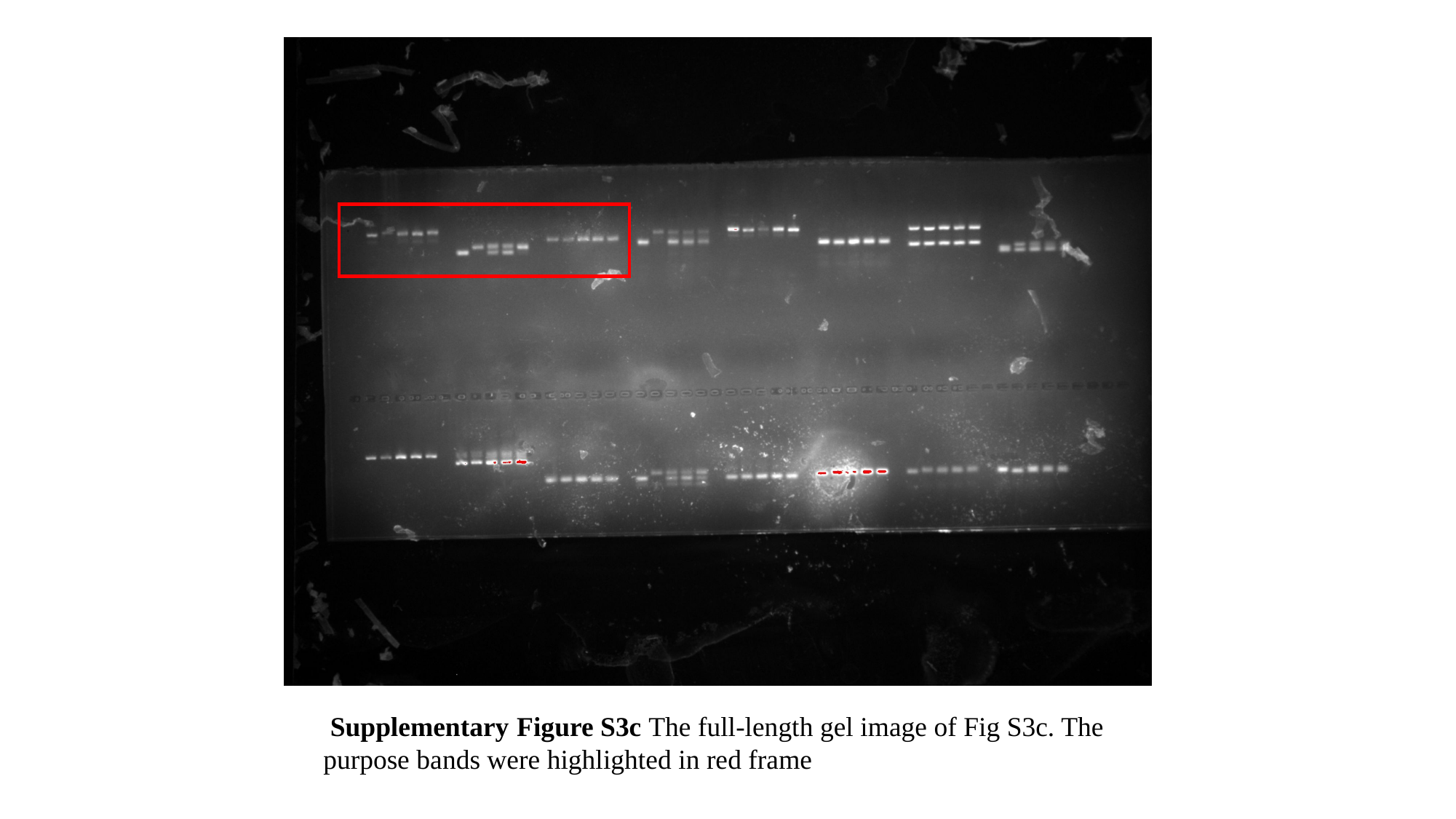

Supplementary Figure S3c The full-length gel image of Fig S3c. The purpose bands were highlighted in red frame
